# Simulating Honey Bee Large‐Scale Colony Feeding Studies Using the BEEHAVE Model—Part I: Model Validation

**DOI:** 10.1002/etc.4839

**Published:** 2020-09-22

**Authors:** Amelie Schmolke, Farah Abi‐Akar, Colleen Roy, Nika Galic, Silvia Hinarejos

**Affiliations:** ^1^ Waterborne Environmental Leesburg Virginia USA; ^2^ Syngenta Crop Protection, Greensboro North Carolina USA; ^3^ Sumitomo Chemical, Saint Didier au Mont d'Or France

**Keywords:** Ecological modeling, Honey bee colony model, Calibration, Validation methods, Model performance indicators, Overwintering losses

## Abstract

In pesticide risk assessments, semifield studies, such as large‐scale colony feeding studies (LSCFSs), are conducted to assess potential risks at the honey bee colony level. However, such studies are very cost and time intensive, and high overwintering losses of untreated control hives have been observed in some studies. Honey bee colony models such as BEEHAVE may provide tools to systematically assess multiple factors influencing colony outcomes, to inform study design, and to estimate pesticide impacts under varying environmental conditions. Before they can be used reliably, models should be validated to demonstrate they can appropriately reproduce patterns observed in the field. Despite the recognized need for validation, methodologies to be used in the context of applied ecological models are not agreed on. For the parameterization, calibration, and validation of BEEHAVE, we used control data from multiple LSCFSs. We conducted detailed visual and quantitative performance analyses as a demonstration of validation methodologies. The BEEHAVE outputs showed good agreement with apiary‐specific validation data sets representing the first year of the studies. However, the simulations of colony dynamics in the spring periods following overwintering were identified as less reliable. The comprehensive validation effort applied provides important insights that can inform the usability of BEEHAVE in applications related to higher tier risk assessments. In addition, the validation methodology applied could be used in a wider context of ecological models. *Environ Toxicol Chem* 2020;39:2269–2285. © 2020 The Authors. *Environmental Toxicology and Chemistry* published by Wiley Periodicals LLC on behalf of SETAC.

## Graphical abstract

Left: Comparison plot of adult bee numbers and honey stores between data from an example large‐scale colony feeding study (LSCFS, dots) and model outputs (shaded areas). Right: Quantitative performance indicators for BEEHAVE across several LSCFSs used for validation. The combination of visual and quantitative validation methods provides comprehensive insight into model performance across studies, endpoints and time periods simulated.

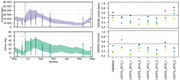

## INTRODUCTION

Potential risks of pesticides to bees are assessed through standardized laboratory studies in which individual adult honey bees or larvae are fed with pesticide‐spiked diets. These lower tier risk assessment studies can be supplemented with higher tier studies that aim to provide progressively more realistic exposure and effects scenarios for honey bee colonies, including semifield studies that combine controlled colony‐level intake of a pesticide with free‐foraging colonies (Oomen et al. 1992; Lückmann and Schmitzer 2019). Published studies vary widely in design, including differences in study duration, beekeeping activities, and exposure of the colonies to a pesticide via feedings of spiked sugar syrup (Faucon et al. 2005; Wu‐Smart and Spivak 2016) or pollen (Sandrock et al. 2014; Dively et al. 2015). Large‐scale colony feeding studies (LSCFSs) conducted for regulatory purposes in the United States include a 6‐wk period of feeding of pesticide‐spiked sugar syrup and the assessment of colony overwintering (Overmyer et al. 2018; Thompson et al. 2019). Variations in their study design make it difficult to statistically compare results between studies. In addition, such studies are very cost and time intensive to conduct, and high overwintering losses of untreated control hives have been observed in some studies. Loss of control colonies indicates that stressors other than pesticides (e.g., resource availability, weather, diseases and/or beekeeping activities) likely influence colony dynamics and overwintering survival, confounding the assessment of impacts caused by pesticides. It is not uncommon that these factors lead to the rejection of individual studies by regulatory agencies as evidence in pesticide risk assessments.

Mechanistic honey bee colony models could provide additional information in the risk assessment process (Becher et al. 2013; Sponsler and Johnson 2017; Kuan et al. 2018). In modeling approaches, study conditions can be fully controlled, allowing for the systematic assessment of factors on study outcomes. Accordingly, honey bee colony models like BEEHAVE (Becher et al. 2014) could be used as tools to inform study design. In addition, mechanistic models could be applied to a wider range of environmental and colony management scenarios than can be addressed in empirical studies.

To be acceptable as tools to complement higher tier risk assessment of honey bees, models need to be thoroughly reviewed and tested (European Food Safety Authority 2014). The European Food Safety Authority (EFSA; 2015) reviewed the BEEHAVE model with respect to its acceptability for use in risk assessment with an overall positive outcome. The lack of the explicit representation of pesticide exposures and effects was identified as the most important impediment to its application in risk assessments. In addition, the EFSA recommended further testing, sensitivity analysis, and validation of the model. In this context, the opinion specifically called for the comparison of BEEHAVE with data from field‐based studies, particularly relying on data from untreated, that is, control, colonies that were not used for model calibration. Such a comparison of outputs from a model to independent empirical data, also referred to as validation, is a pathway to understanding model performance in a context that was not explicitly used in the development and previous testing of the model (Rykiel 1996; Schmolke et al. 2010; European Food Safety Authority 2014).

It is recognized that validation of ecological models applied in risk assessments should be conducted, particularly if data sets for such an exercise are available (European Food Safety Authority 2014). Methods have been discussed in the literature (e.g., European Food Safety Authority 2018), and were recommended to be applied in the context of pattern‐oriented modeling, that is, the assessment of multiple patterns that can be observed in both model and empirical measurements (Grimm and Railsback 2012; European Food Safety Authority 2014). However, quantitative indicators of model performance are not commonly used for complex mechanistic effects models, and there is no consensus about relevant indicators. Comprehensive visual and quantitative model performance analyses are more commonly conducted in other fields of environmental modeling (Harmel et al. 2014), and may inform approaches applied to ecological models.

In the present study, we used the calibrated BEEHAVE model to assess a range of visual and quantitative validation methods described in the literature for various types of environmental models. Accordingly, the validation effort we present can provide insights into validation methods applicable to ecological models in pesticide risk assessments. We present the validation of the BEEHAVE model in the context of LSCFSs. We had access to the untreated control data of 7 LSCFSs conducted in North Carolina (USA) between 2014 and 2017. We used the characterizations of the initial colony conditions, the landscape composition around the study apiaries, the weather, and the feeding of the untreated control colonies to parameterize the model. We used the colony condition data from 2 of the studies for calibration of the model. Data from the remaining 5 studies served as the validation data set. The validation effort specifically focused on assessing BEEHAVE model validity with respect to its ability to 1) predict colony dynamics of untreated controls across the study period dependent on the conditions encountered by the study colonies, and 2) represent varying control colony outcomes due to impacts of external factors (e.g., beekeeping activities).

## MATERIALS AND METHODS

### LSCFSs

For the parameterization, calibration, and validation of the BEEHAVE model, we used untreated control data from LSCFSs conducted in North Carolina (Overmyer et al. 2018; Thompson et al. 2019). In these studies, honey bee colonies were set up in landscapes with low agricultural intensity and were fed untreated sugar syrup (controls) or sugar syrup spiked with different concentrations of a pesticide while being allowed to freely forage in the landscape for nectar and pollen. The studies were conducted following a similar general study design, although the specifics differed between studies. In all studies, colonies were set up from bee packages in the spring, grouped by size into apiaries. Average initial colony sizes varied among studies. Twelve apiaries served as replicates with 2 untreated colonies in each apiary. The transport to the apiary locations corresponded to the study initiation (between late June and early July). The first treatment feeding was applied within 6 d after study initiation. Over a period of 6 wk, 12 treatment feedings were applied to each colony by providing sugar syrup in a feeder integrated in each hive box. The syrup volume provided per feeding differed between studies. In all studies, colonies were also supplied with supplemental (untreated) sugar syrup after the end of the treatment feedings. Feeding timing, frequency, duration and feeding volume and sugar concentration of the syrup supplied during these supplemental feedings varied considerably between studies.

Colony condition assessments (CCAs) were conducted to assess adult bee, egg, larva, and pupa numbers as well as honey and pollen cells using visual estimation of frame coverage (Imdorf et al. 1987). Depending on the study, 4 to 5 CCAs were performed after study initiation and prior to winter. After the overwintering period, 1 to 2 CCAs were conducted between March and April. Data from the CCAs conducted after study initiation were used for comparison with BEEHAVE simulations.

### Uncertainty in empirical data

Visual estimation of frame coverage applied for CCAs is a standard procedure for the assessment of colony condition but does not provide very high precision in the reported measurements (Imdorf et al. 1987). In addition, considerable variability in colony condition occurred within each study despite similar initial setup and size of colonies, as well as near‐identical location (within the same apiary) and hive management. Thus, we estimated the measurement error and between‐colony variability in adult bee numbers and honey stores. Using data from Imdorf et al. (1987) as well as a data set from one LSCFS in which adult bee numbers were assessed with 2 different methods, the measurement error in adult bee numbers was found to be dependent on colony size: more bees in a colony meant larger absolute deviation between 2 colony measurements. From the data presented by Imdorf et al. (1987), we derived that measurements of adult bee numbers in a colony are associated with an error of ±30.7%. Comparable studies for measurements of honey stores were not available. However, estimated volumes of single honey cells vary: with a range of volumes of single cells of 277 to 360 µL, the honey content of a full (capped) honey cell may vary between 382 and 500 mg (Schmickl and Crailsheim 2007; Bush 2011). In addition, a variable percentage of honey cells may not be full, resulting in variable estimates of total honey weight present in a colony based on number of honey cells.

In the comparisons between simulation outputs and data from LSCFSs, we applied ranges to the CCA data to account for the uncertainties just described. The apiary‐specific simulations were compared with the average and the range of subsequent CCAs conducted with the 2 control colonies in the apiary. Adult bee numbers and honey stores were used for comparisons because they had the lowest relative measurement error and were recognized as the most important endpoints of colony health, particularly prior to overwintering (e.g., Genersch et al. 2010; Austin 2014; Döke et al. 2015). This is consistent with the science‐based recommendations for best management practices for beekeepers suggesting that, among other efforts, attempts to reduce overwintering losses should focus on enhancing colony strength and food stores in the fall (Steinhauer et al. 2014). Uncertainty ranges for adult bee numbers and honey stores were estimated from the CCA data of the 2 control colonies in each apiary, resulting in apiary‐ (rather than colony‐) specific ranges of empirical data (observations). We applied the estimated measurement uncertainty to the 2 data points to obtain the lower and upper limit of the apiary‐specific range. Details on the calculations of the ranges of observations are provided in the Supplemental Data (Section 1).

### BEEHAVE model and version

The BEEHAVE model represents processes within a honey bee colony and its interactions with the surrounding landscape through foraging (Becher et al. 2014). The model was implemented in NetLogo (Ver 5.31.; Wilensky 1999) and is publicly available. A local sensitivity analysis was reported along with the first publication of the model (Becher et al. 2014). For the present study, we used the model version BEEHAVE_BeeMapp2015. We applied a few changes to this BEEHAVE version to 1) allow us to reset colonies to the colony conditions from study data on a given date, 2) include the possibility to feed simulated colonies on specified dates with defined amounts of sugar, and 3) set the nursing efficiency of winter bees corresponding to in‐hive bees rather than foragers (which is used as the default in BEEHAVE). In addition, several parameters that were defined in the model code were transferred to the interface of the model to allow the testing of different parameter values. The changes applied to BEEHAVE and the parameter settings applied are described in detail in the Supplemental Data (Section 2). The BEEHAVE version used for the present study, as well as all relevant input files and data, are provided in GitHub in the *Data Availability Statement*.

### Compilation of study‐specific inputs to BEEHAVE

Apiary‐specific input parameter sets were compiled for the BEEHAVE simulations from specifications from the LSCFSs. Inputs include the definition of temporally and spatially explicit bee resource availabilities in the landscapes around the colonies, daily foraging hours determined by weather, initial colony conditions at study initiation, and colony feeding.

#### Landscape

Bee resource availabilities in the landscapes around the hives were derived from land cover data. Information on nectar and pollen availability by crop and seminatural land cover was compiled from the literature. Crops were represented as resources if they were categorized as resources for honey bees by the US Department of Agriculture (2015). For the 5 crops represented (alfalfa, corn, sorghum/millet, soybean, and tobacco), the amount of nectar, including its sugar content, as well as pollen produced by single flowers was estimated using reports from the literature following the approach of Becher et al. (2016) and Schmolke et al. (2019). In addition, flowering period and flower density were determined from literature sources. For all crops providing bee resources, the default BEEHAVE gathering times for nectar and pollen were applied (1200 s for nectar, and 600 s for pollen).

Land covers other than crops provide varying amounts of bee resources for extended time periods. Seasonal resource availabilities are dependent on the land cover type and flowering plant composition. Specific information on floral resources from the range of flowering plants occurring in the region and their association with specific land cover types was not available. Instead, we applied resource categories on a monthly basis to each noncrop land cover type. Bee resource categories were estimated based on data from pollen traps fitted to colonies in the LSCFSs on a few different dates. The estimation of bee resource availability (including nectar and pollen gathering times) in noncrop land covers, particularly in the spring when no data from pollen traps were available, was identified as particularly uncertain, and included in the BEEHAVE calibration (see the *Model calibration* section). Details about the estimation of bee resources in crop and noncrop land cover types are provided in the Supplemental Data (Section 3).

#### Weather

Foraging times available to the simulated foragers per day were derived from weather data. Yearly weather data were retrieved from a single weather station (US National Oceanic and Atmospheric Administration [NOAA] 2018) that was nearest to all apiary locations across the 7 studies. For the determination of foraging hours, BEEHAVE uses the maximum daily temperature and the sunshine hours. However, sunshine hours are not measured routinely by US NOAA weather stations. Instead, we applied the simplifying assumption that no foraging hours were available to bees on days with any reported precipitation, and the full day length was available in case no precipitation was reported for the given day with maximum temperature ≥15 °C. The temperature threshold corresponds to the threshold for foraging used in previous BEEHAVE applications (Becher et al. 2014).

#### Initial colony conditions

We reset the simulated colonies to the measurements reported in each LSCFS at its study initiation date. Study initiation was chosen for the setting of colony conditions in the simulations because colonies newly established from packages may not initially display dynamics comparable to those of established colonies, feeding amounts and locations prior to study initiation were not reported in all studies, and colony transportation may impact colony dynamics in undefined ways (Simone‐Finstrom et al. 2016).

In the simulations, initial colony conditions were set from input files defining the date (day of year) of the study initiation, the presence or absence of the queen (assumed to be present in all colonies), number of eggs, larvae, pupae, and adult workers, honey stores in kg, and pollen stores in g. Honey stores were set assuming that each honey cell contains 500 mg of honey (Schmickl and Crailsheim 2007). A pollen cell was assumed to contain 230 mg of pollen (Schmickl and Crailsheim 2007).

#### Colony feeding

In the LSCFSs, colonies were fed with sugar syrup from top feeders integrated in the hive boxes. Feeding occurred on specified dates, and various volumes of sugar syrup with varying sugar concentrations were provided to the colonies. For the simulation of sugar feeding in BEEHAVE, we applied 2 assumptions: 1) sugar provided in the top feeder represents a direct addition to the existing honey stores of the colony, that is, no foragers are involved in the retrieval of the sugar and the sugar is instantly available for consumption by the colony; and 2) the sugar content of the syrup is decisive of increase in honey stores. Syrup volume and concentration were not considered explicitly but were calculated to reflect the sugar (by weight) in the solution. Feeding schedules listing the date (as day of year) of each feeding event and the sugar fed (as honey equivalent in kg) were compiled as study‐specific input files. Note that in study LSCFS_2016_1, varying quantities of sugar syrup were fed to the colonies in the supplemental feedings, and not all syrup was removed by the bees in all cases. For the BEEHAVE simulations, we applied the highest amount reported per feeding across control colonies.

### Model calibration

The BEEHAVE model was designed based on the mechanistic understanding of a honey bee colony, including many processes along with their parameterizations reflecting a large body of research conducted with honey bees (Becher et al. 2013, 2014). By design, mechanistic models are more flexible and robust for application to conditions not explicitly considered during model development (Stillman et al. 2015) than are statistical models that do not consider system functioning. The calibration of a mechanistic model to a new setting (e.g., geographical and climatic region) should focus on a small subset of parameters that can be assumed to deviate from original assumptions on an ecological basis (Rykiel 1996; Grimm and Railsback 2012).

For the calibration of the BEEHAVE model to data of control colonies in LSCFSs, we focused on a subset of parameters according to the following criteria: 1) BEEHAVE default parameter values that were identified as uncertain (e.g., nectar and pollen gathering times in noncrop land covers); 2) BEEHAVE default parameter values that were not likely to apply to the climatic region where the LSCFSs were conducted (e.g., parameters defining the seasonal egg laying rate); and 3) parameters that likely affect colony dynamics with initial discrepancies between simulations and study data (e.g., nursing efficiency of winter bees). The identified parameters according to these criteria are relevant to 4 submodels in BEEHAVE: egg laying, pollen consumption, brood raising by winter bees, and nectar and pollen availability and quality in the landscape (Table 1).

**Table 1:** BEEHAVE parameters and inputs altered during calibration to achieve improved match between simulation outputs and colony condition assessment (CCA) data from 2 large‐scale colony feeding studies (LSCFSs) used for calibration

Parameter group	BEEHAVE parameter	Values tested during calibration^a^	Parameter description	Reason for inclusion in calibration
Egg laying	egg_laying_x1	150, 175, **385**	Parameters define timing and steepness of seasonal egg‐laying function	Simulated seasonality in egg numbers not matched with CCA data; seasonality of egg laying expected to differ due to climate
	egg_laying_x2	15, 20, **25**		
	egg_laying_x3	32, **36**		
	egg_laying_x4	150, **155**, 187, 200, 220		
	egg_laying_x5	25, **30**, 35, 45, 48, 55		
	MAX_EGG_LAYING	1200, 1400, **1600**, 1800, 1975	Maximum daily egg production	Egg numbers in CCA data highly variable; average exceeded simulated egg numbers
Pollen consumption	DAILY_POLLEN_NEED_ADULT_DRONE	0.0002, **2**	Daily pollen consumption (mg) by life stage/sex	Alternative daily pollen consumption (mg) rates derived from BeeRex model (US Environmental Protection Agency 2014)
	DAILY_POLLEN_NEED_FORAGER	0.041, **1.5**		
	DAILY_POLLEN_NEED_IHBEE	**1.5**, 6.5		
	DAILY_POLLEN_NEED_LARVA	6.53, **23.6**		
	DAILY_POLLEN_NEED_LARVA_DRONE	5.7, **50**		
Brood raising by winter bees	pollenStoreLasting_d	**7**, 14	Defines pollen collection motivation dependent on pollen stores in the hive	Shifting balance between nectar and pollen collection
	WINTERBEE_NURSING	TRUE, **FALSE**	Switch between winter bees performing brood nursing according to in‐hive bees (‘TRUE’) or foragers (‘FALSE’)	Physiological changes reported in winter bees allowing them to revert back to conducting in‐hive tasks efficiently (Winston 1987)
Nectar and pollen availability and quality in the landscape	Forest/woodland spring resource category	Variable resource category scenarios based on flowering estimates (see Supplemental Data, Table S13)	Defines the bee resource availability and gathering times in patches with forest/woodland land cover (see Supplemental Data, Section 3.3)	Estimates for spring resources particularly uncertain; flowering trees potentially provide high resources
	Sugar concentration (mol/L) in nectar from noncrop sources	1, **1.5**	Sugar concentration in nectar	Sugar concentration is variable dependent on plant species, weather, and other factors; 1.5 mol/L reflects high sugar content
	Nectar and pollen availability in noncrop resource categories	Variable availability scenarios dependent on noncrop resource category (see Supplemental Data, Table S14)	Total nectar and pollen available/d from a patch/m^2^ (see Supplemental Data, Section 3.3)	Nectar and pollen availabilities from nonmass flowering crops based on uncertain estimates
	Nectar/pollen gathering times (s) in noncrop resource patches	**1200/600**, 3 variable gathering time scenarios (see Supplemental Data, Table S15)	Gathering times define the time needed by a forager in a resource patch to fill its honey crop/pollen baskets (see Supplemental Data, Section 3.3)	Reports of gathering times vary widely in the literature; gathering times impact resource influx to the colony and forager mortality

^a^ BEEHAVE default values are in bold.

#### Egg laying

The parameters of the egg‐laying function represent the seasonality of egg laying by honey bee queens. Because the seasons are considerably longer in the study area compared with the region for which the egg‐laying function was developed (central Europe; Schmickl and Crailsheim 2007; Becher et al. 2014), we expected a parameter change necessary to successfully represent the LSCFS data.

#### Pollen consumption

Default pollen consumption rates in BEEHAVE differ considerably from the assumptions in the bee risk assessment tool BeeRex used by the US Environmental Protection Agency (2014) due to different sets of literature sources used for parameterization. Because the rates in BeeRex are relevant in the context of bee risk assessments, we tested the corresponding parameter assumptions in the BEEHAVE calibration.

#### Pollen availability over the winter and winter bee nursing

The pollen stores were observed to be lower in the initial simulations compared with the LSCFS data, and low pollen stores over the winter prevented successful brood raising prior to resource availability in the landscape in the model. Overwintering adult bees are represented as foragers in BEEHAVE that have a lower efficiency in caring for brood than in‐hive bees. This assumption was identified as uncertain because winter bees have been shown to experience a change in their physiology that reverts the transition to foragers once brood raising starts again in the late winter (Winston 1987). In the calibration, we tested higher target pollen storage as well as winter bee nursing efficiency set to the same level as in‐hive bees.

#### Resource definitions in the landscape

For noncrop land covers, estimates of nectar and pollen availability, nectar sugar concentration, and gathering times across the season were uncertain. They were assumed to deviate from the BEEHAVE default assumptions about a flower patch corresponding to a mass‐flowering crop. We assumed that land covers with lower resource quality, reflected by the noncrop resource category in the present study, provide lower nectar and pollen availability, lower nectar sugar concentration, and higher gathering times. Several patterns of resource availability were tested (Table 1 and Supplemental Data, Section 4).

The calibration was conducted by assessing the impact of changes in each parameter group on colony dynamics, and the improvement of the seasonal patterns of adult bee numbers, honey stores, brood numbers, and pollen stores compared with CCA data from control colonies from 2 LSCFSs (LSCFS_2015_1 and LSCFS_2015_2). The determination of best match between simulations and CCA data ranges was conducted by visually inspecting graphed apiary‐specific outcomes. In addition, the number of mean BEEHAVE outputs that fell within the apiary‐specific CCA data ranges for the fall (late October) CCA was used for comparison between calibration simulations. Additional details about the calibration are provided in the Supplemental Data (Section 4).

A calibration of BEEHAVE to a specific study type has not been reported previously in the literature. A calibration using quantitative indicators (such as used for the validation in the present study) would require the a priori definition of indicators from the LSCFS data that should be optimized with the model. A large number of simulations would have to be conducted with the model, for example, using a Latin hypercube approach, for which the BEEHAVE model is not currently set up. Instead, the focus of the present study was on the validation of BEEHAVE, and the validation methodology applicable.

### Model validation

With the calibrated BEEHAVE model, we conducted apiary‐specific simulations representing the validation data set (LSCFS_2013_1, LSCFS_2014_1, LSCFS_2014_2, LSCFS_2016_1, and LSCFS_2016_2). In total, untreated control colonies from 59 apiaries were simulated (with 10 repetitions each). Note that one apiary from study LSCFS_2013_1 was excluded because it was reported to be compromised during the conduction of the study. The calibrated BEEHAVE model was applied using apiary‐specific inputs for initial colony conditions (average conditions of the 2 control colonies in each apiary), landscape composition around each apiary, study‐specific sugar feeding schedules, and year‐specific weather. Each apiary‐specific simulation was repeated 10 times with BEEHAVE to capture the stochastic processes in the model, including mortality and foraging (Becher et al. 2014).

#### Visual model performance analysis

For the validation of the model, the apiary‐specific predictions (BEEHAVE simulation outputs) were compared with the observations (adult bee numbers and honey stores reported for the control colonies in the corresponding apiaries after study initiation). The first step in assessing model performance involved visual inspections by graphing observations and predictions together in multiple ways (Ritter and Muñoz‐Carpena 2013; Harmetl et al. 2014; European Food Safety Authority 2018). The graphical representation can give insight into the temporal differences in performance, the potential bias of model outputs, and the distribution of deviations between observations and predictions. We graphed predictions and observations on a timeline whereby we combined the data from the apiaries of each study to limit the number of plots. In addition, we graphed predictions against observations for each study, using the average CCA data (from 2 control colonies) and the average simulation data per apiary. These scatter plots provide an overview of model performance and bias in predictions. Distribution plots of deviations between predictions and observations visualize the distribution and bias of predictions.

#### Model bias

In a second step, systematic over‐ and underprediction (bias) was assessed (Moriasi et al. 2007; Ritter and Muñoz‐Carpena 2013; Harmel et al. 2014). Bias was calculated as the absolute (*b*) and relative (*b*_rel_) difference between apiary‐specific average observation and prediction:

$$b=\;\frac{1}{n}\sum_{i=1}^{n}\left((,P_{i}-O_{i},)\right)$$

$$b_{\mathrm{rel}}=\frac{b}{\overset{®}{O}}$$

where *O* are the observations, *P* the predictions and $\overset{®}{O}$ the mean of the *n* observations.

For the observations, either the means of the 2 reported colony measurements of the 2 control colonies in each apiary were used, or the means of the upper and lower range of the CCA data, as applicable. The absolute and relative bias values were averaged across all CCAs conducted per apiary and across all apiaries in each study.

#### Quantitative model performance analysis

In the third step, quantitative indicators of model performance, or goodness‐of‐fit indicators, were calculated. Various indicators were introduced in the literature. Quantitative indicators provide a concise and comparable measure of model performance, but may indicate different levels of goodness‐of‐fit depending on the data sets analyzed. Thus, it has been recommended to calculate 2 or more indicators for a better understanding of model performance (Moriasi et al. 2007; Bennett et al. 2013; Ritter and Muñoz‐Carpena 2013; Harmel et al. 2014). In Table 2, we list the 3 goodness‐of‐fit indicators that we calculated for apiary‐specific simulations of each study, and across the validation data set. For all 3 indicators, a value of 0 indicates a perfect match between predictions and observations. Currently, no guidance exists on what may constitute a good fit in complex ecological models. Where available, we used threshold values for goodness‐of‐fit indicators proposed for other model types in the literature. For normalized root mean square error (NRMSE), a threshold of 0.5 was proposed for acceptable performance of toxicokinetic‐toxicodynamic models, which simulate toxic effects in individual organisms (European Food Safety Authority 2018). For the RMSE‐standard deviation ratio (RSR), values ≤0.7 were considered acceptable by Moriasi et al. (2007) for deterministic hydrological models. No threshold for acceptable normalized mean absolute error (NMAE) values was suggested in the literature.

**Table 2:** Goodness‐of‐fit indicators and area comparison statistics applied to compare apiary‐specific predictions from BEEHAVE simulations with observations from the validation data set^a^

Goodness‐of‐fit indicator	Equation	Remarks	References
Normalized mean absolute error (NMAE)	$\begin{array}{l} \mathrm{MAE}=\frac{1}{n}\sum_{i=1}^{n}\left|O_{i}-P_{i}\right|\\ \mathrm{NMAE}=\frac{\mathrm{MAE}}{\overset{®}{O}} \end{array}$	Other names used for the same indicator: relative MAE, MARE	Bennett et al. 2013; Harmel et al. 2014
Normalized mean square error (NRMSE)	$\begin{array}{l} \mathrm{RMSE}=\sqrt{\frac{1}{n}\sum_{i=1}^{n}{\left(O_{i}-P_{i}\right)}^{2}}\\ \mathrm{NRMSE}=\frac{\mathrm{RMSE}}{\overset{®}{O}} \end{array}$	Indicator is very sensitive to outliers; NRMSE ≤0.5 suggested acceptable performance for TKTD models (European Food Safety Authority 2018)	Bennett et al. 2013; European Food Safety Authority 2018; Harmel et al. 2014
RMSE‐standard deviation ratio (RSR)	$\mathrm{RSR}=\frac{\mathrm{RMSE}}{\mathrm{STDE}V_{\mathrm{obs}}}=\frac{\sqrt{\sum_{i=1}^{n}{\left(O_{i}-P_{i}\right)}^{2}}}{\sqrt{\sum_{i=1}^{n}{\left(O_{i}-\overset{®}{O}\right)}^{2}}}$	Indicator of how well the model explains the variance in the observations; indicator is sensitive to outliers	Moriasi et al. 2007; Bennett et al. 2013
Adequacy (A)	$A=\frac{\min\left((,\mathrm{UL}\left(P\right),\mathrm{UL}\left(O\right),)\right)-\max\left((,\mathrm{LL}\left(P\right),\mathrm{LL}\left(O\right),)\right)}{\mathrm{UL}\left(O\right)-\mathrm{LL}\left(O\right)}$	Indicator takes a high value if the range of observations falls within the range of predictions	Gabsi et al. 2014; Preuss et al. 2010; Scholten and Van der Tol 1994
Reliability (R)	$R=\frac{\min\left((,\mathrm{UL}\left(P\right),\mathrm{UL}\left(O\right),)\right)-\max\left((,\mathrm{LL}\left(P\right),\mathrm{LL}\left(O\right),)\right)}{\mathrm{UL}\left(P\right)-\mathrm{LL}\left(P\right)}$	Indicator takes a high value if the range of predictions falls within the range of observations	Gabsi et al. 2014; Preuss et al. 2010; Scholten and Van der Tol 1994

^a^ In the equations, *O* are the observations, *P* the predictions, and $\overset{®}{O}$ the mean of the *n* observations. *UL* stands for upper limit and *LL* for lower limit of the ranges of observations and predictions, respectively.

TKTD = toxicokinetic toxicodynamic.

The goodness‐of‐fit indicators were designed to provide insight into the performance of deterministic models compared with empirical data without explicitly considering uncertainty. In the literature, authors recommend the consideration of uncertainty in data (observations) when assessing model performance (Moriasi et al. 2007; Bennett et al. 2013; Harmel et al. 2014). The degree of error inherent in the empirical measurements should inform the criteria for model performance.

For the present study, we established that the uncertainties in the observations (CCA data) were considerable and varied depending on the measured endpoint (see the *Uncertainty in empirical data* section). In addition, BEEHAVE includes stochastic processes, resulting in a range of outputs from identical parameter settings. To represent the uncertainty in the predictions, we calculated the error of the mean of the repeat BEEHAVE simulations (*n* = 10) relative to the range limits in the observations. If the mean of predictions was within the range of observations, no error was assumed in any of the goodness‐of‐fit indicators for the given pairwise comparison of prediction and observation. If a prediction value was lower than the lower limit of the observation range, the error was calculated relative to the lower observation range limit. Correspondingly for predictions larger than the upper observation limit, the error was calculated relative to the upper observation limit. Because both observations and predictions were ranges of values, we also applied area comparison statistics (adequacy and reliability) to the validation data sets (Table 2) as indicators comparing ranges rather than point values. Area comparison statistics have been applied previously for the performance analysis of several ecological models (Scholten and Van der Tol 1994; Preuss et al. 2010; Gabsi et al. 2014). Adequacy and reliability take a value of 1 for a perfect match, and 0 in case of no overlap between observations and predictions.

The goodness‐of‐fit indicators and area comparison statistics were recalculated correcting for study‐specific bias, that is, BEEHAVE simulation outputs were corrected by absolute bias prior to applying indicator calculations. Accordingly, 4 versions of the indicators were calculated by comparing 1) means from apiary‐specific BEEHAVE simulations with CCA data (treating both predictions and observations as deterministic); 2) bias‐corrected means from apiary‐specific BEEHAVE simulations with CCA data; 3) means from apiary‐specific BEEHAVE simulations with CCA data ranges; and 4) bias‐corrected means from apiary‐specific BEEHAVE simulations with CCA data ranges.

## RESULTS

### Model calibration

A comparison of BEEHAVE simulations prior to calibration with the control colony data from the LSCFSs chosen as calibration data sets revealed several discrepancies between simulated and observed data. Egg production by the queen ceased much earlier in the year in BEEHAVE compared with the study colonies. High peak adult bee numbers in late summer and high honey stores simulated in BEEHAVE pointed to overestimation of resource availability in the landscape, or too low foraging effort in collecting the resources. In addition, low pollen stores suggested that the balance between foraging effort for nectar and pollen was not reflective of foraging in the study colonies (see also Supplemental Data, Section 4).

Testing several combinations of the parameter values within a parameter group and across groups (Table 1) resulted in increased match between simulations and CCA data in one endpoint while reducing the match in another endpoint. We concluded that a good match across all endpoints (adult bee and brood numbers, honey and pollen stores) and dates available for comparison may not be attainable. Subsequently, the calibration effort was focused on achieving a good match in adult bee numbers and honey stores. These 2 endpoints were identified as most indicative of colony health (e.g., Genersch et al. 2010; Austin 2014; Döke et al. 2015) and had the lowest uncertainty in data reported from the studies. Fall was identified as a particularly important time of the year because colony condition prior to overwintering is related to subsequent overwinter survival (Abi‐Akar et al. 2020, this issue). The parameter combination that resulted in the best match between apiary‐specific simulations and CCA data with the uncertainty range applied is summarized in Table 3. In Table 3, we also summarize the impact of each parameter group on the simulation outcomes.

**Table 3:** Calibrated parameter values used in BEEHAVE validation simulations, and observed impacts on BEEHAVE simulation outcomes of alterations in parameter values applied in the calibration

Parameter group	BEEHAVE parameter	Calibrated value^a^	Impact of parameter on BEEHAVE simulation outcomes
Egg laying	egg_laying_x1	385	Improved match with data: extension of egg‐laying season led to higher adult bee numbers in the winter and increased spring colony growth
	egg_laying_x2	**15**	
	egg_laying_x3	36	
	egg_laying_x4	155	
	egg_laying_x5	**45**	
	MAX_EGG_LAYING	**1200**	Slightly improved match with data: decrease in maximum egg‐laying rate led to slightly lower adult bee numbers in summer and fall; reduced egg‐laying rate may reflect higher mortality in eggs in study colonies
Pollen consumption	DAILY_POLLEN_NEED_ADULT_DRONE	**0.0002**	Improved match with data: pollen consumption rates derived from BeeRex (US Environmental Protection Agency 2014) led to higher adult bee numbers in the winter and increased spring colony growth
	DAILY_POLLEN_NEED_FORAGER	**0.041**	
	DAILY_POLLEN_NEED_IHBEE	**6.5**	
	DAILY_POLLEN_NEED_LARVA	**6.53**	
	DAILY_POLLEN_NEED_LARVA_DRONE	**5.7**	
Brood raising by winter bees	pollenStoreLasting_d	**14**	Improved match with data: increased pollen collection increased winter bee numbers and spring colony growth
	WINTERBEE_NURSING	FALSE	No improved match with data: the assumption of higher efficiency in brood nursing by winter bees led to more variable brood and adult numbers in the spring but no consistent improvement in match with study data
Nectar and pollen availability and quality in the landscape	Forest/woodland spring resource category	**High spring resource availability**	Improved match with data: assuming only high pollen availability in the spring did not have clear impacts on colony spring growth but assuming high nectar and pollen moderately increased spring growth
	Sugar concentration (mol/L) in nectar from noncrop sources	**1**	Improved match with data: lower sugar concentration in nectar from noncrop resources led to moderate decrease in summer and fall honey stores as well as small decrease in adult bee numbers
	Nectar and pollen availability in noncrop resource categories	Exponential decline in nectar and pollen availability with declining resource category	No improved match with data: increase in resource availability had only very minor impacts on colony dynamics
	Nectar/pollen gathering times (s) in noncrop resource patches	**Variable gathering time scenario with highest difference in gathering times dependent on noncrop resource category**	Improved match with data: considerably reduced adult bee numbers in summer and lower honey stores due to broader range of gathering times dependent on noncrop resource category

^a^ Bold values deviate from BEEHAVE default.

### Model validation

#### Visual model performance analysis

**Figure 1:**
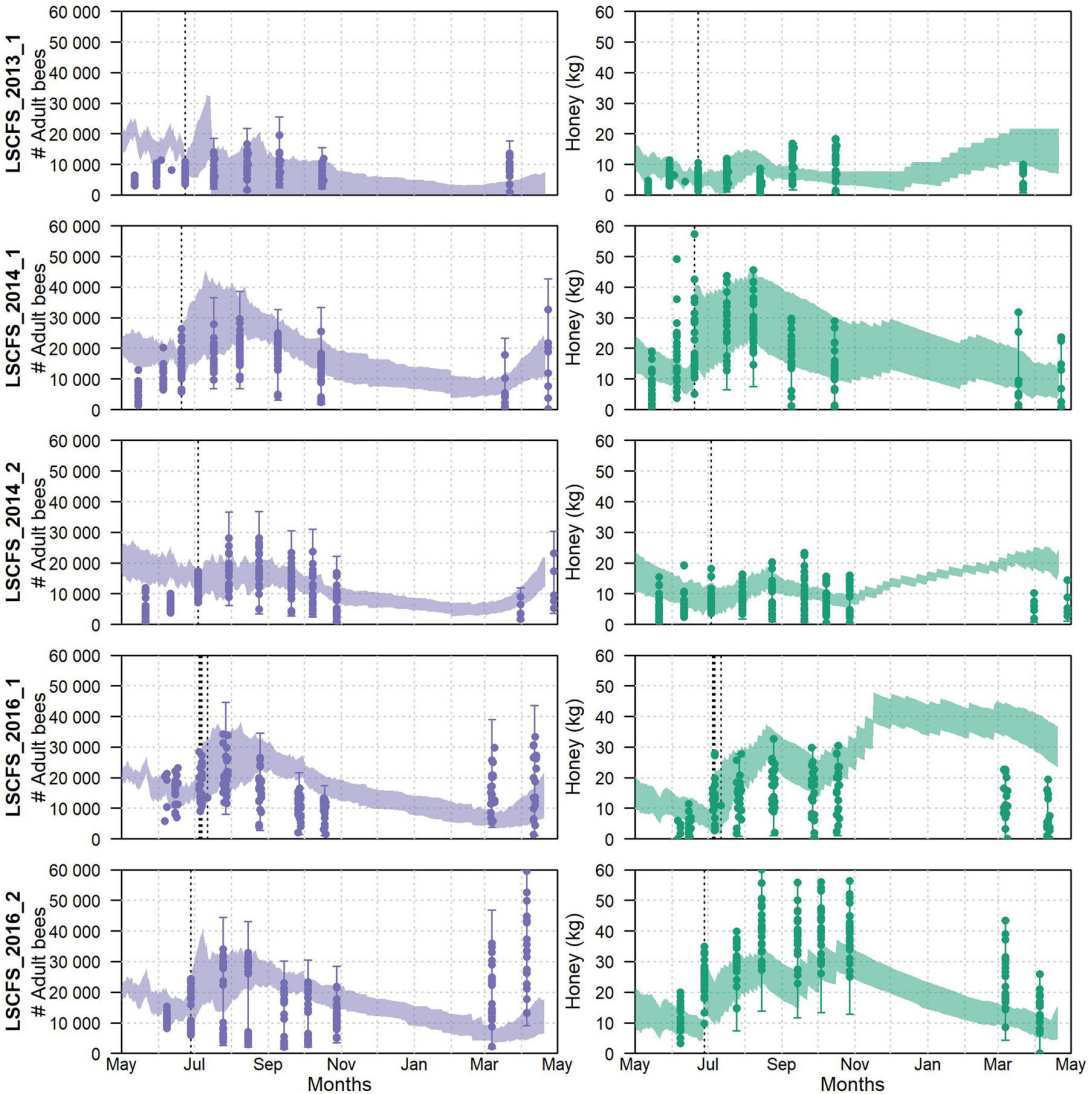
Adult bee numbers (left) and honey stores (right) across large‐scale colony feeding studies (LSCFSs) used for BEEHAVE validation. Shaded areas show the range of BEEHAVE outputs across all apiaries and repetitions simulated (110 simulations for LSCFS_2013_1; 120 simulations for all other LSCFSs). Dots represent the data reported from the colony condition assessments (CCAs). Lines with whiskers mark the range of CCA data across all apiaries and with uncertainty range applied to observations. Vertical dotted lines mark the study initiation. (Note that in LSCFS_2016_1, study initiation occurred over several days across apiaries.)

For the validation of the BEEHAVE model, we first graphed the model predictions (BEEHAVE outputs) against the observations (CCA data) per LSCFS (Figure 1). Temporal dynamics of adult bee numbers and honey stores across the first study year suggest that BEEHAVE captures well the empirical data for the 5 studies. However, simulated adult bee numbers in the spring of the second study year were not captured equally well.

**Figure 2:**
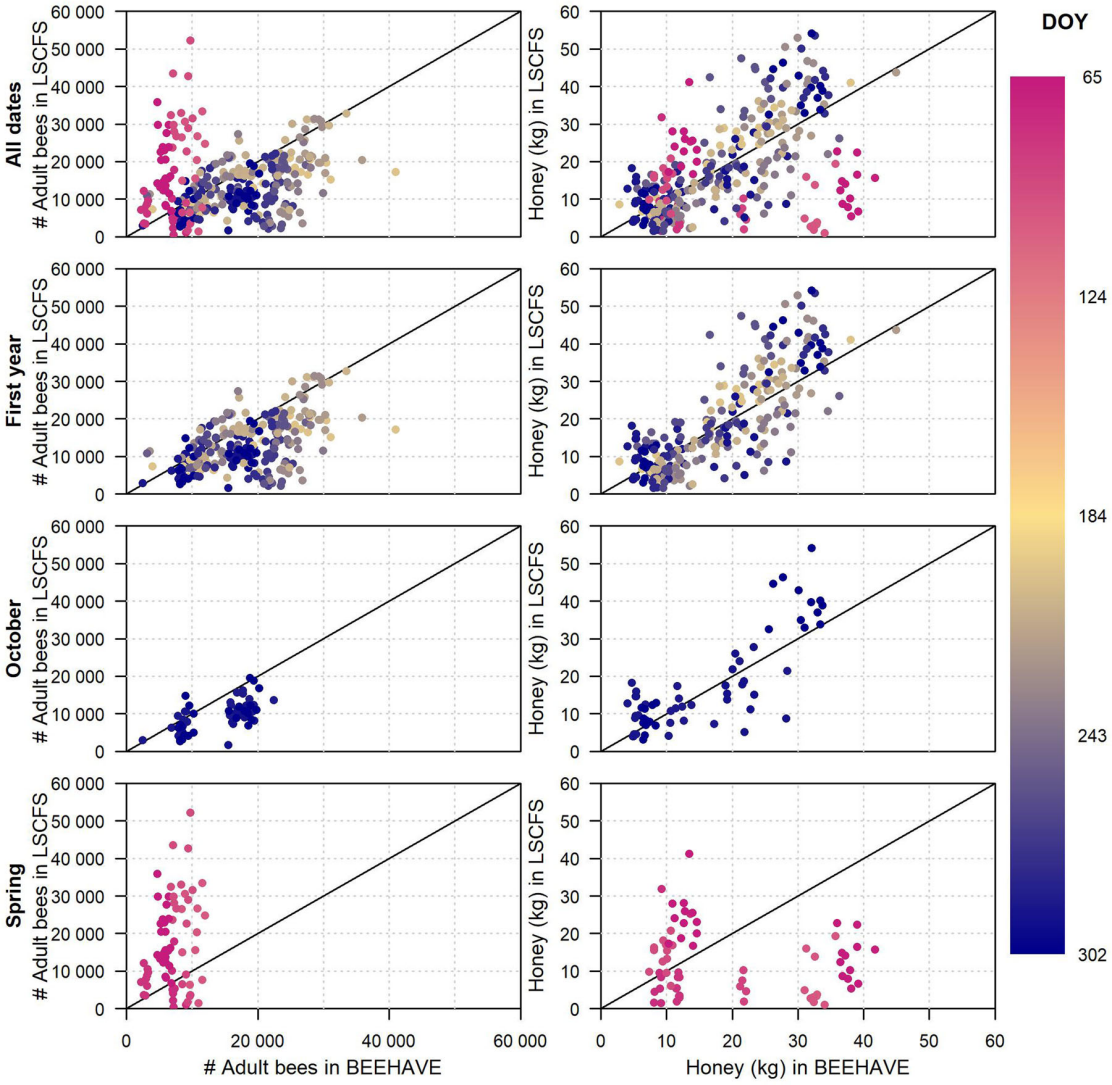
Scatter plots of apiary‐specific colony condition assessment data against BEEHAVE outputs across the 5 studies used for validation. The means of the 2 control colonies/apiary in the studies are compared with the mean outputs from corresponding BEEHAVE simulations (10 repetitions). The black line denotes a perfect match between predictions and observations. DOY = day of year; LSCFS = large‐scale colony feeding studies.

**Figure 3:**
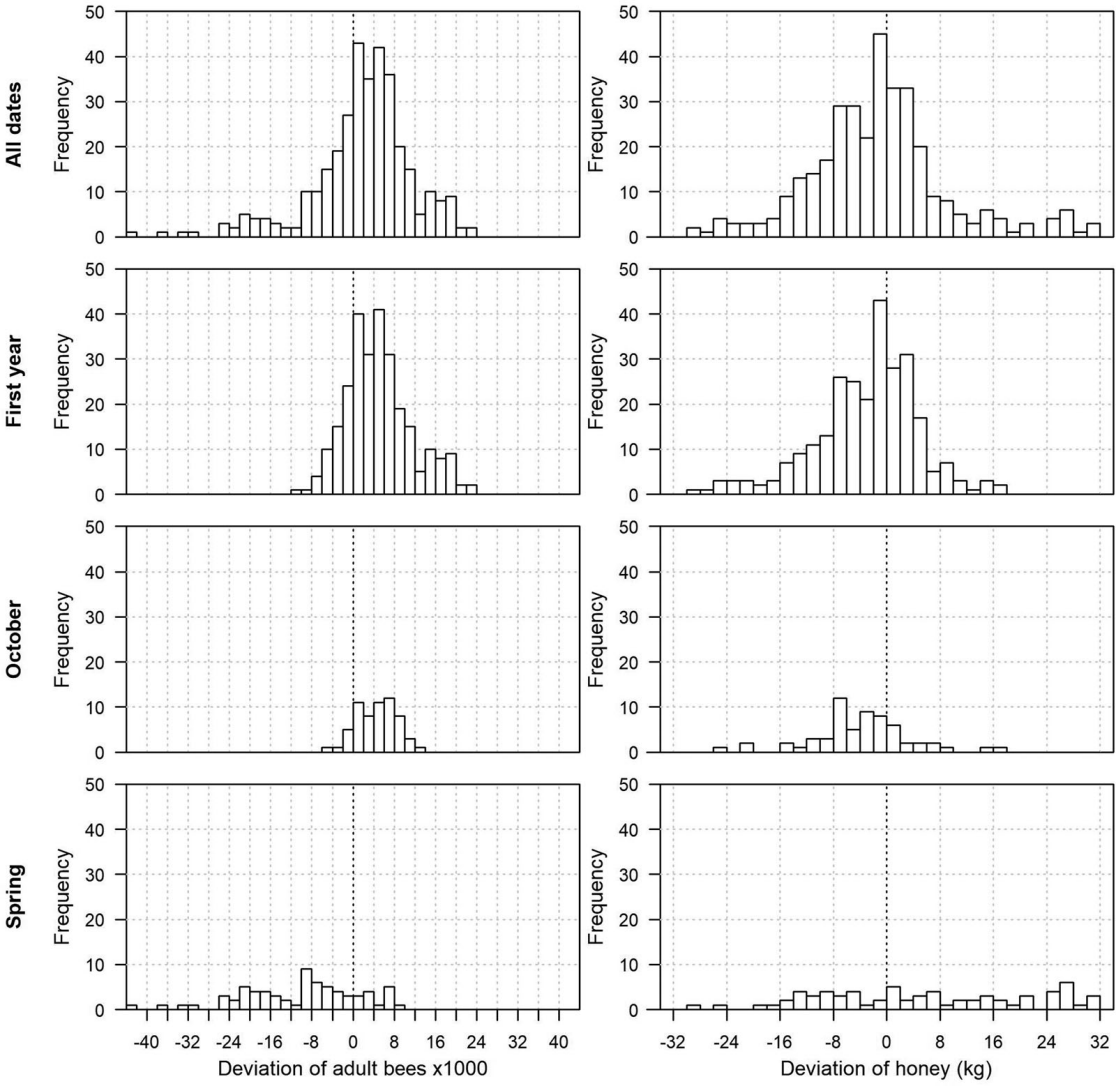
Distribution plots of deviations between apiary‐specific colony condition assessment data and BEEHAVE outputs across the 5 studies used for validation. The means of the 2 control colonies/apiary in the studies are compared with the mean outputs from corresponding BEEHAVE simulations (10 repetitions). Deviations >0 correspond to overestimation by BEEHAVE; deviations <0 correspond to underestimation.

In Figure 2, the means of the 10 repetitions conducted with BEEHAVE were plotted against each mean of the CCA data from the 2 control colonies in the corresponding apiary (no ranges applied to the CCA data). The relationship between apiary‐specific predictions and observations are visualized in these scatter plots. In addition, scatter plots for 3 temporal data subsets are presented: first study year (predictions and observations from 4 dates between 15 July and 2 November), October (from the last date of CCA for each study, between 16 October and 2 November), and spring of the following year (from 1–2 CCAs conducted after overwintering, between 6 March and 29 April). Predictions are scattered around the observations irrespective of absolute adult numbers or honey stores. If the spring period (second study year) was removed, the time of year also did not seem to influence the match between prediction and observation. In Figure 3, the distribution of deviations between predictions and observations (predictions minus observations) is shown for the whole data set as well as the temporal subsets. Apart from the CCAs from the spring of the second study year, the deviations appear to be normally distributed. The mean of the distributions for honey was close to 0, suggesting no bias in predictions of honey stores by BEEHAVE simulations. In contrast, distributions of deviations for adult numbers are shifted to positive values, indicating that BEEHAVE simulations systematically overpredict adult bee numbers (bias).

From the visual model performance analysis, the spring period was identified as a subset of the data for which BEEHAVE does not provide accurate quantitative predictions. Accordingly, the quantitative model performance analysis was focused on predictions and observations from the first study year. Bias in the predictions was considered explicitly.

#### Model bias

**Figure 4:**
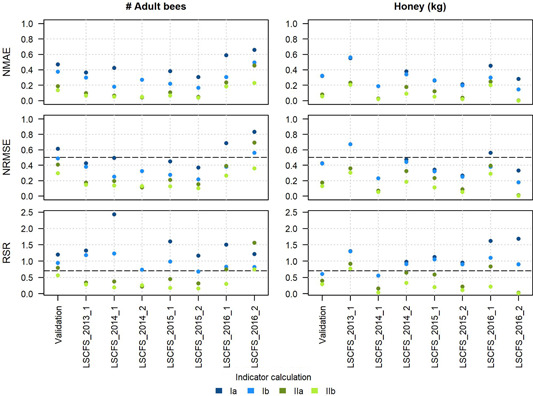
Goodness‐of‐fit indicators for adult bee numbers (left) and honey stores (right) comparing first‐year BEEHAVE outputs with large‐scale colony feeding study (LSCFS) data across the validation data set, and by study (including studies used for calibration). Indicator calculation: Ia: colony condition assessment (CCA) data (apiary‐ and CCA‐specific averages) compared with BEEHAVE outputs (average/not corrected for bias); Ib: CCA data (apiary‐ and CCA‐specific averages) compared with BEEHAVE outputs (average/bias‐corrected). IIa: CCA data (apiary‐ and CCA‐specific ranges) compared with BEEHAVE outputs (average/not corrected for bias); IIb: CCA data (apiary‐ and CCA‐specific ranges) compared with BEEHAVE outputs (average/bias‐corrected). Thresholds for NRMSE and RSR proposed for other model types are shown as dashed horizontal lines. NMAE = normalized mean absolute error; NRMSE = normalized mean square error; RSR = RMSE‐standard deviation ratio.

Across the validation data set, BEEHAVE tended to overpredict adult bee numbers (Table 4). For the honey stores, no considerable bias was obvious across the validation data set (−5% bias), but study‐specific biases varied considerably. Because in many cases bias for both endpoints exceeded the recommended level of 5% (Harmel et al. 2014), we calculated the quantitative goodness‐of‐fit indicators with and without bias corrected. Goodness‐of‐fit indicators, particularly the RSR are sensitive to bias, potentially leading to the rejection of a model that otherwise correctly predicts patterns in the empirical data (Ritter and Muñoz‐Carpena 2013). In Figure 4, goodness‐of‐fit indicators are presented with and without correction for bias, demonstrating the impact of bias on these indicators.

**Table 4:** Bias in simulated adult bee numbers and honey stores compared with first‐year colony condition assessment data by study^a^

	Adult bee numbers		Honey stores (kg)	
Study	Absolute bias	% bias	Absolute bias	% bias
LSCFS_2013_1	1587	19	–0.4	−5
LSCFS_2014_1	7209	43	0.2	1
LSCFS_2014_2	−79	0	1.4	17
LSCFS_2016_1	8032	57	6.4	41
LSCFS_2016_2	8610	62	−10.6	−28

^a^ Positive numbers denote overpredictions by BEEHAVE, and negative numbers correspond to underpredictions.

LSCFS = large‐scale colony feeding study.

#### Quantitative model performance analysis

We compared the MAE of the predictions with the corresponding observations, normalized to the average of the observations (NMAE), the NRMSE, and the RSR (Figure 4). The goodness‐of‐fit indicators are shown for each individual study and across the validation data set for the first year of the studies. Previously suggested thresholds for acceptable model performance are included in the plots for comparison.

Goodness‐of‐fit indicators were improved by comparing BEEHAVE simulations with CCA data ranges (representing the uncertainty in the empirical data) rather than treating the CCA data as deterministic (no range applied). Considerable variation in goodness‐of‐fit indicators was observed between studies for all 3 indicators applied. Considering the whole validation data set (“validation” in Figure 4), BEEHAVE showed a better performance predicting honey stores in the colonies compared with adult bee numbers. Correcting for bias was important for the studies in which high bias was observed (e.g., LSCFS_2016_2).

When calculated compared with the range of observations, NRMSE values for adult bees were ≤0.5 for the first year with the exception of study LSCFS_2016_2, which had the largest bias (see Table 4). When bias was corrected, NRMSE also fell below the threshold for this study. For honey stores, NRMSE values all fell below the threshold compared with the observation range.

Relative to the threshold suggested by Moriasi et al. (2007), RSR values suggest lower performance ratings of BEEHAVE than the previous indicators (NMAE and NRMSE). However, when RSR was calculated comparing BEEHAVE outputs with the uncertainty range of the CCA data and correcting for bias, the indicator was below the suggested threshold of 0.7 for most studies. Two cases of RSR for individual studies indicate a model performance indicator above the threshold even after considering the range in the CCA data and correcting for bias: simulated adult bee numbers in LSCFS_2016_2, and honey stores in LSCFS_2013_1.

**Figure 5:**
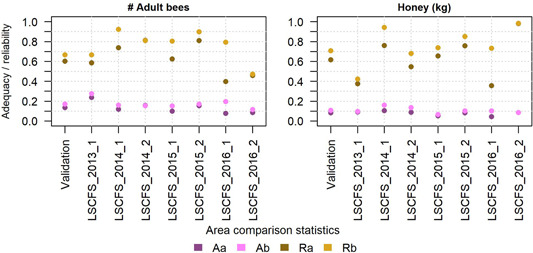
Area comparison statistics for adult bee numbers (left) and honey stores (right) comparing first year BEEHAVE outputs with large‐scale colony feeding study (LSCFS) data across the validation data set, and by study (including studies used for calibration). Aa = adequacy, not corrected for bias; Ab = adequacy, bias‐corrected; Ra = reliability, not corrected for bias; Rb = reliability, bias‐corrected.

Considering area comparison statistics, the adequacy of the model predictions of adult bees as well as honey stores for the first year was low for the whole validation data set as well as for individual studies (A <0.25 for most studies; Figure 5). Correcting for bias did not improve model adequacy considerably in most cases. Model reliability was 0.6 across all validation studies for adult bee numbers in the first year. Reliability in this endpoint improved to 0.67 if bias was corrected (Figure 5). For honey stores, the first‐year validation data set comparison indicated a model reliability of 0.62 (0.71 with bias corrected). The discrepancy between adequacy and reliability can be explained by smaller ranges of predictions compared with ranges of observations.

## DISCUSSION

The validation exercise presented in the present study provides important insights that can inform the usability of BEEHAVE in applications related to higher tier pesticide risk assessments. Although BEEHAVE was developed based on multiple literature sources reporting mainly on honey bee data from experimental studies conducted in Great Britain and Germany (Becher et al. 2014), the model is capable of closely simulating patterns of untreated control colony dynamics observed in LSCFSs conducted in North Carolina (southeastern USA; e.g., Overmyer et al. 2018; Thompson et al. 2019). This finding is far from trivial. The BEEHAVE model is mechanistic, that is, it represents multiple interacting processes of the complex functioning of a colony based on current understanding of honey bee biology. Still, the model is a simplification of the real system (as is any model) and may not capture multiple processes influencing honey bee colonies in the field. Processes and variables not included in the model may have been deemed of lesser importance for the observable dynamics of a single colony during model development or may not be understood in the scientific literature. Thus, it cannot be assumed that the model can simulate colony dynamics in varying climates, study designs, or environmental conditions. Instead, the transferability of the model to such new contexts has to be demonstrated.

Prior to validation, we calibrated BEEHAVE using untreated control data from 2 of the 7 available LSCFSs. We did not attempt to calibrate the model across the large number of parameters included in BEEHAVE, but instead focused on a small subset of parameters that we identified as important based on longer foraging seasons in the southeastern United States compared with Central Europe, uncertainties in landscape resource inputs, and observed deviations between initial simulations (prior to calibration) and study data. A calibration including more (or all) parameters in the model, the definition of realistic ranges of parameters, and the a priori definition of target model performance in various endpoints and time points may have led to a closer match between simulation outputs and data used for calibration. However, we were aiming to conduct the validation with a limited calibration effort that would be feasible in future applications. We focused on the applicability of BEEHAVE to LSCFSs without extensive additional analysis of the model and its calibration, and how validation methodologies can be applied to honey bee colony models in particular and complex ecological models in general. Still, we would like to note several insights we gained about the model during the calibration. Notably, using the pollen consumption rates assumed by the US Environmental Protection Agency (2014) instead of the BEEHAVE default assumptions improved the match between simulated colony dynamics and study data. In addition, uncertainties relating to resource availability, represented by longer gathering times than used in default BEEHAVE in patches not containing mass‐flowering crops, led to a better match between simulations and study data. Accordingly, field data on nectar and pollen gathering times in different land cover types could improve parameterization of the model and the assessment of landscape composition with respect to colony dynamics and may reduce the calibration effort.

For the validation, we applied the calibrated model to apiary‐specific conditions from 5 studies that were not included in the calibration effort, and thus can be seen as independent data sets within the context of LSCFSs conducted in North Carolina. Visual and quantitative validation methods suggest that the calibrated BEEHAVE model provides good predictions of adult bee numbers and honey stores observed in colonies across the first year of each study. However, predictions for the following spring were not reliable.

A good correspondence between data from colony field studies and BEEHAVE simulations has also been reported by Agatz et al. (2019). Using visual comparisons between simulation outputs and study data, the authors were able to show that dynamics of adult bee numbers and brood were reproduced by BEEHAVE. As in the present study, landscape and weather were represented in detail according to the location of the bee hives and their surroundings. However, the field study and corresponding simulations did not include overwintering, so the performance of BEEHAVE in simulating spring colony dynamics was not assessed in their study (Agatz et al. 2019). Further evaluation of BEEHAVE with respect to overwintering and spring colony dynamics may be necessary if it were to be used for the accurate assessment of colony dynamics spanning into a second study year.

Evaluation of goodness‐of‐fit to each LSCFS revealed that the variability in model performance was dependent on the study, because studies differed from each other in the specifics of design and field conditions. In addition, variability in observed colony conditions within each study was also larger than in the BEEHAVE simulations. The variability in study data likely has multiple sources that are not all fully captured by the model. Uncertainties of the CCA methods used in the LSCFSs are documented in the literature (Imdorf et al. 1987; Delaplane et al. 2013). We used the documented uncertainties and available data from one study to assess variability from this source. Uncertainty in empirical CCA data is important to consider when one is assessing model performance. The remaining variabilities between colonies experiencing very similar conditions and management point to factors influencing colony dynamics that are beyond what can be reasonably controlled in a field‐based study. Large variability in outcomes of overwintering success (Abi‐Akar et al. 2020, this issue) further suggests that factors may be influencing colony dynamics and overwintering success in LSCFSs that are currently not well understood. Such factors and processes are not captured in BEEHAVE because the model is based on current knowledge about honey bee colonies, and on initial conditions and other factors reported in the studies.

The improvement in model performance indicators if bias was corrected points to a higher accuracy in the prediction of temporal dynamics compared with absolute colony endpoints by the model. Adult bee numbers were overpredicted by BEEHAVE for most studies. Depending on the time of day when the CCAs were conducted in the studies (not reported), it is possible that adult bees were undercounted because a subset of bees was actively foraging (Imdorf et al. 1987). Mortality rates of the developmental bee stages assumed in BEEHAVE could also contribute to this observed consistent discrepancy.

Validation of ecological models has been recognized as important to demonstrate the acceptability of a model for use in an intended context (Rykiel 1996; Schmolke et al. 2010; European Food Safety Authority 2014). However, a methodology applicable to validation of ecological models remains elusive. If comparisons between model outputs and empirical data are reported, they are commonly limited to a visual comparison. Although visual comparison between empirical observations and model predictions are recognized as an indispensable step in model validation, the combination with quantitative validation methods provide more comprehensive and less subjective insights into model performance (Elliott et al. 2000; Bennett et al. 2013). Various quantitative indicators have been applied to specific ecological models (Elliott et al. 2000; Higgins et al. 2001; Preuss et al. 2010; Gabsi et al. 2014; Ashauer et al. 2016; Focks et al. 2018), and the European Food Safety Authority (2018) has suggested quantitative indicators for the validation of the general unified threshold model of survival (GUTS) with laboratory toxicity data. However, no common approach for model performance analyses and validation has been introduced for use across ecological models. In other fields, particularly hydrological modeling, quantitative model performance analyses are more common, and comprehensive approaches to model validation have been described (Moriasi et al. 2007; Bennett et al. 2013; Ritter and Muñoz‐Carpena 2013; Harmel et al. 2014). We adapted available validation approaches from the literature and applied them to assess model performance of BEEHAVE across apiary‐specific simulations.

The visual methods applied provide a comprehensive overview of patterns in the predictions compared with observations (e.g., with respect to temporal system behavior). These methods proved necessary in the present study to assess model performance simulating colony dynamics in different seasons. Because ecological systems and models may be driven by different processes depending on season and/or environmental triggers, model performance may vary across different time periods. Timeline plots (compare to Figure 1) are necessary for the first analysis of patterns. Scatter plots (Figure 2) provide additional important information because they can summarize the comparison between observations and model predictions depending on each data point. These graphical representations also provide important tools to compare model performance across different endpoints (e.g., adult bee numbers and honey). Multiple endpoints are commonly considered in empirical ecological data sets, and ecological models usually provide multiple outputs. Assessing model performance across different endpoints increases our understanding of model behavior and can identify endpoints that are most accurately predicted. In summary, visual model performance analyses can support model validity for qualitative applications such as comparison of scenarios, but are limited in assessing the accuracy of quantitative model predictions.

Quantitative indicators provide a systematic way of comparing model performance across multiple empirical data sets such as observation data sets from multiple studies. In addition, quantitative indicators could be used to compare performances of different models used to simulate the same empirical data. The validation methodology applied to BEEHAVE could inform validation methods in a wider context of ecological models (Schmolke et al. 2010; European Food Safety Authority 2014). We explicitly addressed the uncertainty in the empirical data prior to comparison with model outputs. Measurement error and, particularly, variability in empirical data were identified as substantial in the context of CCAs. Ecological field data in general may come with more variability than other environmental data (such as hydrological data), and the analysis of uncertainties in field measurements may need to be given higher focus. Accordingly, quantitative indicators need to be based on a thorough understanding of the model and the patterns it can simulate because quantitative indicators alone may be misleading if they are applied without considering temporal and other sources of variability in empirical data sets as well as model outputs (Ritter and Muñoz‐Carpena 2013). Therefore, we recommend that quantitative indicators should always be presented in the context of visual model performance analyses.

Bias in model predictions should be considered because patterns or seasonal dynamics are generally considered more important to be reproduced by ecological models than exact values in single endpoints and time points (e.g., Grimm and Railsback 2012; Stillman et al. 2015). Bias correction can help isolate discrepancies caused by a lack of prediction of temporal dynamics rather than consistent over‐ or underprediction (Harmel et al. 2014). However, the variability in bias observed in the comparison with the 5 LSCFSs used for validation of BEEHAVE suggest that validation results should always be presented without correcting for bias first.

The goodness‐of‐fit indicators as used in the present study may need to be reviewed for application to ecological models including stochastic processes and their comparison with data that are represented by a range. We used the goodness‐of‐fit indicators to compare the means of repeat simulations with BEEHAVE with both the means and ranges of apiary‐specific CCA data. For comparison of data ranges, normalized error calculations (such as NRMSE and NMAE) may be more informative than error measures addressing the variance in the observations (RSR; other comparable indicators have been suggested in the literature; Ritter and Muñoz‐Carpena 2013). Previously suggested thresholds for NRMSE and RSR may not be applicable across ecological models. Instead, model performance criteria based on quantitative indicators may be developed for a given model or ecological application that reflect the objectives of the application (Bennett et al. 2013).

In contrast to goodness‐of‐fit indicators, area comparison statistics are designed for the comparison of 2 data ranges corresponding to the range of repeat simulations with BEEHAVE, and the CCA data range representing the uncertainty in the empirical data. However, the limitations of area comparison statistics may also need to be considered because they do not consider the absolute values compared, that is, the increase in data ranges with increase in mean values is not accounted for. In addition, these indicators suggest complete lack of model adequacy and reliability if observation and prediction ranges do not overlap, irrespective of how far apart the 2 data sets are. This causes a particular problem with the indicators if both compared ranges are small, that is, in case of precise predictions and observations.

During calibration and validation of ecological models, it should be further considered that these models are often highly complex and include stochastic processes. Accordingly, large sets of simulations for model calibration and validation may be very time intensive if all parameters, their possible ranges, and the stochasticity of a model are to be fully captured. The extent of calibration and validation effort should be informed by its objectives in terms of expected model precision as well as its representation of variability in empirical study data.

## CONCLUSIONS

In the present study, the availability of control data from 7 LSCFSs presented a unique opportunity for the assessment of the BEEHAVE model. The studies represent a distinct study design that was not considered previously with BEEHAVE. This also applies to the geographical (and climatic) region (North Carolina) where all studies were conducted. We used data from 2 LSCFSs for calibration of the model, and the remaining 5 for validation. The detailed visual and quantitative performance analyses suggested that the calibrated BEEHAVE model provides good agreement with apiary‐specific data across the first study year in the 5 validation study data sets. However, model outputs did not match with observed colony conditions after overwintering, assessed in the following spring. The visual and quantitative validation methods applied may be useful to assess performance of ecological models in general whereby quantitative indicators should always be applied in combination with visual methods. We highlight the considerations necessary to address variability and uncertainty in empirical data for model validation as well as model stochasticity.

We recommend the calibrated BEEHAVE model as useful tool to predict colony dynamics in LSCFSs prior to overwintering. A dependence on study conditions including weather, landscape, initial conditions, and feeding can be further explored with the model. Simulated outcomes are typically less variable than observations from colonies in the field. Accordingly, predictions of colony sizes (number of adult bees and honey stores) from BEEHAVE should not be used as precise predictions of properties of individual colonies, but as predictions of trends across colonies that experience similar conditions. In that respect, the model can be used to estimate the relative impact of the different conditions on colony outcomes at different times of the first year. For instance, conditions and colony managements from the different studies can be addressed systematically, and recommendations for study designs resulting in defined outcomes (e.g., prior to overwintering) can be derived (Abi‐Akar et al. 2020, this issue).

Beyond the context addressed in the present study, the successful validation of first‐year dynamics of LSCFSs suggests that the model could also be a useful tool to simulate pesticide exposure and effects if a pesticide module becomes available and standard scenarios are established (Schmolke et al. 2019). The suitability for the application to other field study designs and geographical and climatic regions is also likely. However, calibration for such altered contexts of application would still be necessary, and context‐specific validation recommended.

## Supplemental Data

The Supplemental Data are available on the Wiley Online Library at https://doi.org/10.1002/etc.4839.

## Supporting information

This article includes online‐only Supplemental Data.

Supporting information.

## Data Availability

Data, associated metadata, and calculation tools are available from the corresponding author (schmolkea@waterborne-env.com). The model code, inputs and data analysis scripts are provided on GitHub: https://github.com/Waterborne-env/BEEHAVE-LSCFS-Application.
