# Supplementary material for: Simulating Honey Bee Large‐Scale Colony Feeding Studies Using the BEEHAVE Model—Part I: Model Validation
Source: Environ Toxicol Chem. 2020 Sep 22;39(11):2269–85. doi: 10.1002/etc.4839 (PMC7702171; doi:10.1002/etc.4839)
Supplement: Supplementary file 1 — Supporting information. [file ETC-39-2269-s001.pdf]

# Simulating honey bee large-scale colony feeding studies using the BEEHAVE model. Part I: model validation

Supplemental Material

Author: Amelie Schmolke

## 1. Representation of uncertainty in CCA data

In the comparisons between simulation outputs and data from LSCFS, we applied ranges to the CCA data to account for the uncertainties in the empirical data originating from measurement errors in visual estimation of frame coverage and variability between control colonies in the same apiary. We applied the uncertainty ranges according to the following rules:

1. **Adult bee numbers**
   a. Between-colony variability: for each apiary and CCA, the two colonies' reported adult numbers served as the minimum and maximum uncertainty range.
   b. Measurement error: the lower and upper limits of the range were defined using the range of measurement error (±30.7%) derived from Imdorf et al. (1987); lower limit: MIN(adult bee number) × 69.3%; upper limit: MAX(adult bee number) × 130.7%.
2. **Honey stores**
   a. Between-colony variability: for each apiary and CCA, the two colonies' reported honey stores served as the minimum and maximum uncertainty range.
   b. Measurement error: the lower limit of the range was calculated by using the lower estimate of honey weight per capped honey cell (382 mg). In uncapped honey cells, an average honey content of 50% was assumed. The percentage of uncapped honey cells depends on the time of year: 66.8% of honey cells were assumed uncapped in summer and fall, and 100% were assumed uncapped in early spring. Accordingly, the lower limit of honey stores (kg) was calculated as:

      <u>Summer and fall (day-of-year > 196):</u>

      $$(\text{MIN(honey cells)} \times 0.668 \times 382 \times 10^{-6}\ \text{kg} \times 0.5) + (\text{MIN(honey cells)} \times (1 - 0.668) \times 382 \times 10^{-6}\ \text{kg}).$$

      <u>Spring (day-of-year < 121; all honey cells assumed uncapped):</u>

      $$\text{MIN(honey cells)} \times 382 \times 10^{-6}\ \text{kg} \times 0.5$$

      The upper limit of the range was calculated by applying the higher estimate of honey weight in a capped honey cell (500 mg) to all cells (uncapped and capped). The higher number of the two CCA measures was multiplied by this weight to obtain the estimated honey store in kg:

      $$\text{MAX(honey cells)} \times 500 \times 10^{-6}\ \text{kg}$$

## 2. Changes to the BEEHAVE model and parameter settings

In order to represent study-specific details with BEEHAVE and to apply changes to default BEEHAVE parameters for calibration, we had to apply a few changes to the code of the model (version "BEEHAVE_BeeMapp2015.nlogo"). The adapted BEEHAVE version and input files used for the current project is provided on GitHub (https://github.com/Waterborne-env/BEEHAVE-LSCFS-Application).

In the studies, colonies were set up from bee packages in the spring of the first year of the study. Colonies were fed for a few weeks, then moved to the final locations of the study apiaries shortly prior to the first treatment feeding. To simulate the colony conditions observed in the studies at study initiation, BEEHAVE colonies had to be set to the defined conditions on the date of study initiation. Setting of colony condition (defining adult bee, egg, larva, pupa numbers as well as honey and pollen stores) on a given date was made possible by adding the option for an input file and a corresponding procedure to BEEHAVE. The added procedure was implemented corresponding to the existing procedure in BEEHAVE ("ReadBeeMappFileProc") but with extended functionality. The name of the new input file is set by "AssessmentFile" in the project-specific BEEHAVE version. The file is used by the model if "ReadAssessmentFile" is switched on. If such an input file is set, the new procedure "AssessmentCorrectionProc" is called. On the date (or rather, day of year) defined in the input file, the colony is reset to the listed number of eggs, larvae and pupae. For each brood stage, the proportion of worker to drone brood present prior to colony reset is preserved. The number of worker eggs is evenly assigned to each age cohort of eggs (i.e., across the three age cohorts reflecting 1-, 2-, and 3-day-old eggs). The same strategy is applied to the other brood stages for worker and drone brood. The adult bee number listed in the input file is used to reset the adult worker numbers in BEEHAVE. From the updated brood numbers, the number of needed in-hive bees is calculated, which ensures sufficient nursing of present brood. After assigning the number of in-hive bees from the number of workers defined in the input file, the remaining number of adult workers is assigned to be foragers. For the adult workers, the age structure (bees per age cohort or forager squadron, respectively) is retained from the age structure in the simulated colony prior to application of the assessment file. Adult drone numbers are not changed by the input file. Honey stores and pollen stores in the colony are represented as homogeneous stores in BEEHAVE, and are reset to the weight defined in the input file.

In the LSCFSs, specific feeding schedules were applied to the colonies. Feeding was defined by the date and sugar amount fed. No honey was removed (harvested) from the colonies during the study. With the input file "FeedingScheduleFile" in the project-specific BEEHAVE version, feeding amounts on given dates could be set. In the BEEHAVE procedure "BeekeepingProc", honey is added to the colony's store on the defined dates according to the input file.

For the purpose of altering BEEHAVE default parameters in the context of calibration, a few parameters were added to the BEEHAVE interface for the study-specific model version. These parameters were defined in the code of the BEEHAVE model previously, and thus, were not accessible for systematic changes by the user (the user would need to change the code to set alternative parameter values). By defining the parameters and their assigned values on the model interface, they can be changed between simulation runs as needed. The parameters added to the interface are listed in Table S1. In addition, we included the possibility to change the nursing efficiency of winter bees ("WINTERBEE_NURSING"). In BEEHAVE, winter bees are

represented as foragers. Foragers in the model are less efficient in nursing brood, i.e., one forager per brood item is needed to ensure development of all brood. In contrast, each in-hive bee can care for three brood items at a time. If winter bee nursing is switched on the project-specific model version, winter bees are able to care for three brood items as well. Parameter values applied in the validation simulations are listed in Table S2.

**Table S1.** BEEHAVE model parameters moved from the code to the interface. Interface parameters can be changed between simulation runs without changing the model code.

| Parameter name | Default value | Description |
|---|---|---|
| MAX_EGG_LAYING | 1600 | Maximum rate of eggs laid by the queen in one day |
| egg_laying_x1<br>egg_laying_x2<br>egg_laying_x3<br>egg_laying_x4<br>egg_laying_x5 | 385<br>25<br>36<br>155<br>30 | Parameters of the function defining seasonal egg laying; the function follows the HoPoMo model by Schmickl and Crailsheim (2007) |
| DAILY_POLLEN_NEED_ADULT_DRONE<br>DAILY_POLLEN_NEED_FORAGER<br>DAILY_POLLEN_NEED_IHBEE<br>DAILY_POLLEN_NEED_LARVA<br>DAILY_POLLEN_NEED_LARVA_DRONE | 2<br>1.5<br>1.5<br>23.6<br>50 | Daily pollen consumption rates in mg. Note pollen consumption rates include the pollen-equivalent of jelly consumption in larvae |
| pollenStoreLasting_d | 7 | Defines pollen collection motivation dependent on pollen stores in the hive |

**Table S2.** Interface parameter settings applied in calibrated BEEHAVE. Apiary-specific input files can be found on GitHub (https://github.com/Waterborne-env/BEEHAVE-LSCFS-Application). Parameters are listed in alphabetical order.

| Parameter name | Parameter value | Remarks |
|---|---|---|
| AddedPollen_kg | 0.5 | Unused: no pollen added |
| AddedPollen | FALSE | |
| AllowReinfestation | FALSE | |
| AlwaysDance | FALSE | |
| ContinuousBroodRemoval | FALSE | |
| CRITICAL_COLONY_SIZE_WINTER | 1 | Threshold of colony winter death switched off |
| DAILY_POLLEN_NEED_ADULT_DRONE | 0.0002 | Parameter value from BeeRex |
| DAILY_POLLEN_NEED_FORAGER | 0.041 | Parameter value from BeeRex |
| DAILY_POLLEN_NEED_IHBEE | 6.5 | Parameter value from BeeRex |
| DAILY_POLLEN_NEED_LARVA | 6.53 | Parameter value from BeeRex |
| DAILY_POLLEN_NEED_LARVA_DRONE | 5.7 | Parameter value from BeeRex |

| Parameter name | Parameter value | Remarks |
|---|---|---|
| DANCE_INTERCEPT | 0 | |
| DANCE_SLOPE | 1.16 | |
| details | TRUE | Unused: write file not used |
| DotDensity | 0.01 | Unused: default experiments not used |
| DroneBroodRemoval | FALSE | |
| EfficiencyPhoretic | 0.115 | Unused: mites not simulated |
| EfficiencyPhoretic2 | 0 | Unused: mites not simulated |
| EggLaying_IH | TRUE | |
| egg_laying_x1 | 385 | Calibrated |
| egg_laying_x2 | 15 | Calibrated |
| egg_laying_x3 | 36 | Calibrated |
| egg_laying_x4 | 155 | Calibrated |
| egg_laying_x5 | 45 | Calibrated |
| Experiment | none | |
| FeedBees | FALSE | |
| FrameType | Shallow frame | Unused: not a model parameter |
| HarvestingDay | 135 | Unused: honey harvest not simulated |
| HarvestingPeriod | 80 | Unused: honey harvest not simulated |
| HarvestingTH | 20 | Unused: honey harvest not simulated |
| HiveType | Langstroth | Unused: not a model parameter |
| HoneyHarvesting | FALSE | |
| HoneyIdeal | FALSE | |
| KillAllMitesInCells | FALSE | |
| KillAllMitesInCells2 | FALSE | |
| KillOpenBrood | FALSE | |
| KillOpenBrood2 | FALSE | |
| MAX_BROODCELLS | 108000 | Specific to LSCFS studies |
| MAX_EGG_LAYING | 1200 | Calibrated |
| MAX_HONEY_STORE_kg | 54 | Specific to LSCFS studies |
| MAX_km_PER_DAY | 7299 | |
| MergeColoniesTH | 5000 | Unused: colony merging not simulated |
| MergeWeakColonies | FALSE | |
| MiteReinfestation | 0.1 | Unused: mites not simulated |
| MiteReproductionModel | Martin | Unused: mites not simulated |
| modelledInsteadCalcDetectProb | FALSE | Unused: detection probability defined in INPUT file |
| N_INITIAL_BEES | 10000 | |
| N_INITIAL_MITES_HEALTHY | 0 | |
| N_INITIAL_MITES_INFECTED | 0 | |
| PollenIdeal | FALSE | |
| pollenStoreLasting_d | 14 | Calibrated |

| **Parameter name** | **Parameter value** | **Remarks** |
|---|---|---|
| ProbLazinessWinterbees | 0 | |
| QueenAgeing | FALSE | |
| RAND_SEED | 27, 41, 9, 43, 17, 3, 21, 11, 28, 6 | Random number seeds used for 10 repetitions of each apiary-specific simulation set |
| ReadAssessmentFile | TRUE | Setting of apiary-specific initial colony condition from file |
| ReadBeeMappFile | FALSE | |
| ReadFeedingSchedule | TRUE | Setting of study-specific feeding schedule from file |
| ReadInFile | TRUE | Setting of apiary-specific landscape resources from file |
| RemainingHoney_kg | 5 | Unused: honey harvest not simulated |
| RemovalDay1 | 100 | Unused: honey harvest not simulated |
| RemovalDay2 | 140 | Unused: honey harvest not simulated |
| RemovalDay3 | 180 | Unused: honey harvest not simulated |
| RemovalDay4 | 220 | Unused: honey harvest not simulated |
| RemovalDay5 | 240 | Unused: honey harvest not simulated |
| SeasonalFoodFlow | TRUE | Unused: food flow defined in INPUT file |
| ShowAllPlots | TRUE | Interface presentation |
| SQUADRON_SIZE | 100 | |
| stopDead | TRUE | |
| Swarming | No swarming | |
| TIME_NECTAR_GATHERING | 1200 | Unused: patch-specific gathering times defined in INPUT file |
| TIME_POLLEN_GATHERING | 600 | Unused: patch-specific gathering times defined in INPUT file |
| TreatmentDay | 270 | Unused: mite treatment not simulated |
| TreatmentDay2 | 0 | Unused: mite treatment not simulated |
| TreatmentDuration | 40 | Unused: mite treatment not simulated |
| TreatmentDuration | 0 | Unused: mite treatment not simulated |
| VarroaTreatment | FALSE | |
| Virus | DWV | Unused: mites not simulated |
| Weather | Weather File | Setting of year-specific weather from file |

| Parameter name | Parameter value | Remarks |
|---|---|---|
| WINTERBEE_NURSING | FALSE | Parameter added during calibration |
| writeFile | FALSE | |

# 3. Compilation of BEEHAVE input files defining bee resource availability in the landscape

## *3.1. Land cover composition*

The landscape composition differed between study apiaries. In BEEHAVE, resource availability in the landscapes around the simulated colonies is represented by patches of spatially homogeneous availability of nectar and pollen. In an input file to the model, resources (patches) in the landscape are defined on a daily basis by several characteristics: 1) the distance of the patch from the hive, 2) the total amount of nectar, 3) the sugar concentration in the nectar, 4) the total amount of pollen, 5) the time needed by a forager to fill her honey stomach with nectar (nectar gathering time), and 6) the time needed to fill her pollen baskets (pollen gathering time).

The resource definition depends on the land cover in each patch. We retrieved the landscape composition from the Cropland Data Layer (CDL; US Department of Agriculture 2016) around each apiary location and study year for a radius of 1.5 km. It was assumed that the land cover composition did not change during the duration of a single study, i.e. the same composition of land covers was present around a given apiary location from June of the year of study initiation through April of the following year when the study was completed.

CDL information gives the land cover in every 30×30 $m^2$ pixel of the landscape based on satellite data. For the current study, we defined a patch in the BEEHAVE input as an area of adjacent pixels with identical land cover in the CDL data for all land covers other than deciduous forest. Apiary locations with low area coverage of row crops were chosen in the studies to minimize potential contamination of the study bee colonies with pesticide exposures from the surrounding landscape. As a result, the landscapes around the apiary locations were dominated (to various extents) by deciduous forest. In order to avoid patches represented in the BEEHAVE input from becoming too large, deciduous forest land cover was split up into patches along a super-imposed grid. Each grid cell covered 25 ha, limiting all deciduous forest patches represented in the landscape input files to a maximum area of 25 ha.

The distance between each patch and the colony was calculated using the centroid of each patch in relation to the apiary location. The resource availability was estimated for a $m^2$ of each land cover type, i.e., nectar and pollen amount available in the patch on a given day was calculated by multiplying the estimated nectar and pollen amount per $m^2$ with the patch area.

## *3.2. Methods for estimating bee resources from crop land covers*

A honey bee colony will experience variable resource levels in the landscape around the bee hive depending on time of year, species and densities of flowers present in the landscape. To set a simulated bee colony in a specific and realistic landscape context, the daily resources in terms of pollen and nectar availabilities have to be estimated.

In BEEHAVE, resources around the simulated bee colony are defined by several parameters. The landscape is assumed to be subdivided into distinct "flower patches." Within each patch, pollen and nectar availability is assumed to be uniform (though variable in time). Flower patches are defined by their distance to the hive and their area. These parameters are constant for each patch. In addition, the total volume of nectar in each flower patch is defined for each day (in liters/$m^2$), the sugar concentration of the nectar (in mol/l) and the amount of pollen (in kg/$m^2$). The time it takes bees to gather a full crop of nectar (50 µl) or a full load of pollen (both pollen baskets filled: 15 mg) is also defined for each patch as nectar and pollen gathering times, respectively. Gathering times do not include the travel time from the colony to the flower patch and back, but only the time a bee is assumed to spend within a patch. For a real forager bee, gathering times for pollen and nectar are dependent on several factors that may include flower density, flower morphology, pollen or nectar amount available from a single flower and other factors. For BEEHAVE, the gathering times were estimated for each flower patch. As a BEEHAVE default estimate, 1200 s are assumed as gathering time for nectar, and 600 s for pollen. These estimates are based on average observed gathering times reported by Winston (1987). Gathering (or foraging trip) times reported in the literature range widely and may exceed the estimates used in BEEHAVE considerably (Dosselli et al. 2016; Eckert et al. 1994; Schmid-Hempel and Schmid-Hempel 1987; Thompson et al. 2016; Winston 1987).

For the landscape resource input to BEEHAVE, we assumed that identical land cover types within the same geographical region provide the same resource levels per area. In the following, we describe the method for compiling bee resource specifications for crop land covers around apiary sites in North Carolina. In Table S3, all crops occurring within a radius of 3 km of apiary sites across two studies conducted in 2015-2016 are listed. No additional crops occurred in relevant land cover area around apiary sites (with 1.5 km radius) in the other five studies and study years available for the current project. We used the classification of crops as bee resources provided by the USDA (2015). Crops that were classified as not attractive to honey bees with respect to nectar or pollen were excluded from representation in BEEHAVE landscape resource input. Additionally, we excluded crops that occurred with very low land cover area because CDL comes with a high uncertainty of land cover classification for very small areas and regionally rare crops.

**Table S3.** Crops present within 3 km radius around apiary sites of studies LSCFS_2015_1 and LSCFS_2015_2.

| Crop (according to CDL) | Attractiveness according to USDA 2015 (pollen/nectar) | Represented in BEEHAVE input | Remarks |
|---|---|---|---|
| Alfalfa | +/++ | Yes | |
| Barley | -/- | No | |
| Canola | +/+ | No | Very low land cover area |
| Corn | +/- | Yes: pollen only | Pollen found in samples from pollen traps collected in LSCFS |
| Cotton | -/+ | No | Very low land cover area |
| Dbl Crop Barley/Corn | | Yes: as corn | |
| Dbl Crop Oats/Corn | | Yes: as corn | |

| Crop (according to CDL) | Attractiveness according to USDA 2015 (pollen/nectar) | Represented in BEEHAVE input | Remarks |
|---|---|---|---|
| Dbl Crop Soybeans/Oats | | Yes: as soybean | |
| Dbl Crop WinWht/Corn | | Yes: as corn | |
| Dbl Crop WinWht/Sorghum | | Yes: as sorghum | |
| Dbl Crop WinWht/Soybeans | | Yes: as soybean | |
| Herbs | +/+ | No | Resource availability would be dependent on type of herb; very low land cover area |
| Millet | +/- | Yes: as sorghum | Pollen found in samples from pollen traps collected in LSCFS (specified as "sorghum/millet") |
| Misc Vegs & Fruits | N/A | No | Not enough information; very low land cover area |
| Oats | -/- | No | |
| Other crops | N/A | No | Not enough information |
| Peaches | +/+ | No | Very low land cover area |
| Rye | -/- | No | |
| Sod/Grass Seed | +/- | No | Low land cover area; however, grass pollen found in low quantities in study pollen samples; we assume they originated from grasses in non-crop land covers |
| Sorghum | +/- | Yes: pollen only | Pollen found in samples from pollen traps collected in LSCFS (specified as "sorghum/millet") |
| Soybeans | +/+ | Yes | Pollen found in samples from pollen traps collected in LSCFS |
| Squash | +/+ | No | Very low land cover area |
| Sweet Corn | +/- | Yes: as corn | |
| Sweet potatoes | +/+ | No | According to USDA (2015): "Propagated vegetatively, and requires pollination only for breeding. Small % of acreage." |
| Tobacco | +/- | Yes: pollen only | Pollen found in samples from pollen traps collected in LSCFS |
| Triticale | -/- | No | |
| Winter Wheat | -/- | No | |

We estimated resource availability from each crop represented in BEEHAVE input by using estimates of nectar and pollen production per flower reported in the literature and extrapolating to resource availability per $m^2$ and day. This methodology of bee resource estimation follows the

methodology introduced by Becher et al. (2016). The following values are required to create BEEHAVE resource patch specifications for each crop:

- Daily nectar production (µl/flower)
- Nectar sugar concentration (mol/l)
- Daily pollen production (mg/flower)
- Flowering period
- Daily flowering density (flower/$m^2$)

This information was compiled from multiple sources and is listed in Table S4 for alfalfa, Table S5 for corn, Table S6 for sorghum (which is also used to represent millet), Table S7 for soybean and Table S8 for tobacco. In the scientific publications reviewed, nectar production by flowers was reported using different units and appropriate measures had to be calculated (volume per flower, and sugar concentration in mol/l). For all crop land covers that provide bee resources, the default BEEHAVE gathering times for nectar and pollen were applied (1200 s for nectar, 600 s for pollen).

**Table S4.** Bee resource specifications for alfalfa (*Medicago sativa*).

| | **Value used for BEEHAVE input** | **Uncertainty range** | **References** |
|---|---|---|---|
| Nectar production per flower and day (µl) | 0.145 µl | 0.29 +/- 0.24 µl per flower reported [1]; nectar production assumed to occur over 2 days as peak nectar production reported to occur over 1-2 days [2] | [1] Cane et al. 2011; [2] Southwick 1984 |
| Nectar sugar concentration (mol/l) | 1.25 mol/l | Value uncertain: [1] report 1070 +/- 986 µg/ µl total sugar in nectar which would correspond to 3.13 mol/l; we use the reported sucrose concentration instead (427 +/- 452 µg/µl); [2] estimate of 40% sugar in nectar (corresponds to 1.17 mol/l) | [1] Cane et al. 2011; [2] Southwick 1984 |
| Pollen production per flower and day (µg) | 0.038 mg | [1] 3326 +/- 845 pollen grains per flower; 90mg bee provision contain 33 +/- 5% pollen and 1.3 million pollen grains; [2] note that 2 days of pollen production per flower are assumed. | [1] Cane et al. 2011; [2] Southwick 1984 |
| Flowering Period | 1 June – 27 September | Period reported for ND and WY applied; exact dates added (flowering period given as June – September) | [3] Dittberner and Olson 1983[1] |
| Flower density during flowering period (flowers/m$^2$) | 4984 flowers/m$^2$ | [4] Average 203 plants/m$^2$ (for spring planting, range: 103 - 319); 275 racemes per plant and year (range: 31-782); 5 flowers per raceme (estimate, no quantitative data found); [3] flower period of 112 days; [2] single flower is open for 2 days; flower density is assumed to be uniform across flowering period. | [4] Bagavathiannen 1999 |

[1]Reference given by: https://www.fs.fed.us/database/feis/plants/forb/medsat/all.html#BOTANICAL%20AND%20ECOLOGICAL%20CHARACTERISTICS

**Table S5.** Bee resource specifications for corn (*Zea mays*)

| | **Value used for BEEHAVE input** | **Uncertainty range** | **References** |
|---|---|---|---|
| Nectar production per flower and day (µl) | -- | Corn does not produce nectar. | USDA 2015 |
| Nectar sugar concentration (mol/l) | -- | | USDA 2015 |
| Pollen production per flower and day (µg) | 430 mg | Uncertainties stem from calculation steps: [1], [2] range of pollen grains per tassel reported; [3] weight of single pollen grain; [1] flowering period, as it is assumed that a tassel produces pollen uniformly across flowering period of 13 days. | [1] Uribellarea et al. 2002; [2] Jarosz et al. 2005; [3] Babendreier et al. 2004 |
| Flowering Period | 22 July – 3 August | Ranges given: 19 - 31 July to 26 July- 7 August | Oberhauser 2001 |
| Flower density during flowering period (flowers/$m^2$) | 8 flowers (tassels)/$m^2$ | From planting density of corn; one tassel per plant assumed; variation is low. | |

**Table S6.** Bee resource specifications for sorghum (*Sorghum* spp.)

| | **Value used for BEEHAVE input** | **Uncertainty range** | **References** |
|---|---|---|---|
| Nectar production per flower and day (µl) | -- | Sorghum does not produce nectar. | Odoux et al. 2012 |
| Nectar sugar concentration (mol/l) | -- | | |
| Pollen production per flower and day (µg) | 116.8 mg | Using information from different sources: [1] no. of pollen grains per inflorescence; [2] pollen size (given in µm with a range, pollen weight calculated assuming density of water); [3] flowering duration (given as range of days) | [1] Prieto-Baena et al. 2003; [2] Reddi and Reddi 1986; [3] Gerik et al. 2003 |
| Flowering Period | 1 – 8 August | Using information from different sources: [1] flowering duration (given as range of days); [2] flowering timing (highly dependent on planting time; by early August in WI) | [1] Gerik et al. 2003; [2] Carter et al. 1989 |
| Flower density during flowering period (flowers/$m^2$) | 27 inflorescences/$m^2$ | Range of 100000-120000 plants per acre given (single inflorescence per plant) | Carter et al. 1989 |

**Table S7.** Bee resource specifications for soybean (*Glycine max*)

| | **Value used for BEEHAVE input** | **Uncertainty range** | **References** |
|---|---|---|---|
| Nectar production per flower and day (µl) | 0.034 µl | Calculated from measured sugar mass produced per flower and sugar concentration, assumption of 2 days anthesis period of single flower | Chiari et al. 2005 |
| Nectar sugar concentration (mol/l) | 0.67 mol/l | +/- 10%; measured from honey crops of bees (rather than from nectar collected directly from flowers) | Chiari et al. 2005 |
| Pollen production per flower and day (µg) | 0.019 mg | Pollen grain per anther: average from [1] and [2]; estimate of number of anthers per flower (10); weight of single pollen grain estimated from pollen diameter (as presented in [1]) | [1] Koti et al. 2004; [2] Palmer et al. 1978 |
| Flowering Period | 5 July – 2 August | May vary by soybean strain planted; time of planting; climate/weather | USDA NASS |
| Flower density during flowering period (flowers/$m^2$) | 370 flowers/$m^2$ | Uncertain: derived from planting density; estimate of number of pods per plant; % flowers producing pods. | [1] |

[1] http://corn.agronomy.wisc.edu/Crops/Soybean/L001.aspx

**Table S8.** Bee resource specifications for tobacco (*Nicotiana tabacum*)

| | **Value used for BEEHAVE input** | **Uncertainty range** | **References** |
|---|---|---|---|
| Nectar production per flower and day (µl) | -- | Tobacco does not produce nectar. | USDA 2015 |
| Nectar sugar concentration (mol/l) | -- | | |
| Pollen production per flower and day (µg) | 60.3 mg | Using information from different sources: [1] average or 45000 pollen grain per anther; [2] pollen grain roughly 0.04 mm in diameter; 5 anthers per flower [1] | [1] Horner and Mott 1979;<br>[2] Cheung et al. 2000 |
| Flowering Period | 15 Sep – 31 Oct | General information: flowering in late summer to mid fall [2]; pollen trapped in Oct | |
| Flower density during flowering period (flowers/m$^2$) | 20 flowers/m$^2$ | 50 cm spacing between plants [2]; assumption of 5 flowers with pollen open per plant and day (no data found) | |

[1]http://www.flora.sa.gov.au/efsa/lucid/Solanaceae/Solanaceae%20species/key/Australian%20Solanaceae%20species/Media/Html/Nicotiana.htm
[2]http://davesgarden.com/guides/pf/go/208243/

***Nectar calculations***

Depending on the publication, measures of nectar production were reported as nectar volume (our target measure), nectar mass, sugar concentration either in % (weight sugar per weight solution) or in weight sugar per volume solution (g/l), or sugar mass. Measures of sugar concentration and their combination with sugar mass were used to calculate sugar concentration in mol/l and nectar volume:

$$\boldsymbol{V = \frac{m_B}{\left(\frac{s}{100}\right)\rho}}$$

where
$V$: volume of solution [l]
$\rho$ : density of solution [g/l]
$m_B$: mass of sugar [g]
$s$: sugar content (% w/w)

For the calculations of sugar concentrations (and nectar volume), we assumed that all sugar occurs in the form of sucrose and used the densities of sucrose solutions as stated in Table S9. Sucrose mass can be expressed as $\mathrm{m_B = n_B\ M_B}$ where $n_B$ is the number of mols and $M_B$ the molar mass of sucrose (342.3 g/mol). For the BEEHAVE input, sugar concentration in nectar has to be provided in mol/l.

**Table S9.** Densities of sucrose solutions. Density values were selected based on a stated percentage of sugar in solution. If the percentage value did not exactly match, the nearest reference value was chosen. Values are compiled and verified from online sources[1].

| **Sucrose content *s* (% w/w)** | **Density *ρ* (g/ml)** | **Sucrose concentration (mol/l)** |
|---|---|---|
| 25 | 1.104 | 0.806 |
| 30 | 1.127 | 0.988 |
| 35 | 1.151 | 1.177 |
| 40 | 1.176 | 1.374 |
| 45 | 1.203 | 1.582 |
| 50 | 1.230 | 1.797 |
| 55 | 1.258 | 2.021 |
| 60 | 1.286 | 2.254 |
| 65 | 1.316 | 2.499 |
| 70 | 1.347 | 2.755 |
| 75 | 1.379 | 3.021 |

Note that the density of water is $\rho = 1$ g/ml; honey has an assumed sucrose content of 75%.

[1] Online sources of sucrose solution density measures:
http://www.lclane.net/text/sucrose.html
http://homepages.gac.edu/~cellab/chpts/chpt3/table3-2.html
http://wiki.houptlab.org/wiki/Density_of_Sugar_Solutions

### *3.3. Estimation of bee resources from non-crop land covers (semi-natural vegetation covers and mixed flower land covers)*

Land covers other than crops provide resources for bees varying over the season dependent on the land cover type and flowering plant composition. Specific information on floral resources from the range of flowering plants occurring in the region and their association with specific land cover types was not available. A subset of honey bee hives in the LSCFSs were fitted with pollen traps on a few different dates between mid-June and mid-October. The source plant species (or genus) of the bee collected pollen were identified, and the percentage of each pollen type per trapping event was determined. We assigned the identified flowering plant species to one of the non-crop land cover types (Table S10) using the habitat information available from a wildflower guide (Thieret et al. 2001). The more plant species per land cover type, the higher the resource availability was assumed. Resource availability was estimated on a monthly basis, i.e., resource availability (nectar and pollen) was assumed to be constant for each month of the year, but variable between months for each land cover type. Resource availability from November through May was estimated using resource categorizations applied in other studies (Frankl et al. 2005; Hines and Hendrix 2005; Lonsdorf et al. 2009). Bee resources from non-floral sources (e.g., honeydew and extrafloral nectaries) were not considered.

The land cover types "barren" and "open water" from CDL were assumed not to provide honey bee resources at any time of the year. All other non-crop land cover types present around study apiary sites were grouped into more general land cover types because the available data and information did not allow for more detailed assumptions. Grouped land covers are listed in Table S10.

**Table S10.** Non-crop land covers grouped for the estimation of honey bee resource availability from the land covers across the year.

| Land cover group | CDL land covers combined |
|---|---|
| Forest/woodland | Deciduous forest, evergreen forest, mixed forest, woody wetland |
| Non-crop agriculture | Fallow/idle cropland, pasture, grassland, other hay/non-alfalfa |
| Open natural | Herbaceous wetland |
| Developed | Developed/open space, low intensity, med intensity, high intensity |
| Shrubland | Shrubland |

We defined five categories of resource availability that could occur in a non-crop land cover patch (Table S11). The lowest category 0 reflects no resource availability. The highest category 5 corresponds to high nectar and pollen availability from flowering plants, corresponding to flowering oilseed rape (Becher et al. 2016). Intermediate categories were assigned with linearly decreasing resource availability from high to low. Resource availabilities per area as well as assumed gathering times were subject to calibration because the estimates were identified as both uncertain and potentially influential to colony dynamics. Initially, BEEHAVE default gathering time assumptions across all resource categories were assumed. During calibration, gathering times varying by resource category were applied.

**Table S11.** Honey bee resources assumed to be available from a land cover assigned with a category for BEEHAVE input. Resource category definitions subject to calibration highlighted in green.

| Category | Sugar concentration (mol/l) | Nectar (l/m$^2$) | Pollen (g/m$^2$) | Nectar gathering time (s) | Pollen gathering time (s) |
|---|---|---|---|---|---|
| 0 | 1.5 | 0 | 0 | 0 | 0 |
| 1 | 1.5 | $2.5 \times 10^{-4}$ | 0.25 | 1200 | 600 |
| 2 | 1.5 | $5 \times 10^{-4}$ | 0.5 | 1200 | 600 |
| 3 | 1.5 | $7.5 \times 10^{-4}$ | 0.75 | 1200 | 600 |
| 4 | 1.5 | $1 \times 10^{-3}$ | 1 | 1200 | 600 |

Based on the number of flowering plant species assigned to each land cover group and their flowering times, resource categories were assigned to non-crop land covers (Table S12). Resource categories were assumed to vary between months of the year. Note that no pollen samples were collected during the studies between November and May. Accordingly, the estimates of resource availability in the early spring were identified as particularly uncertain. It is likely that flowering trees provide high nectar and pollen to bees during this period. Accordingly, the resource category assignment to forest/woodland land cover types during this period was included as a parameter set in the calibration. It was assumed that no resources are available to bees in the landscapes in January and December.

**Table S12.** Assignment of honey bee resource categories by land cover group and by how many of the five most commonly observed flowering plants (that provide bee resources) are flowering at the same time. Resource category assignments subject to calibration are highlighted in green.

| Land cover group | Feb | Mar | Apr | May | Jun | Jul | Aug | Sep | Oct | Nov |
|---|---|---|---|---|---|---|---|---|---|---|
| Forest/woodland | 2 | 3 | 3 | 2 | 1 | 1 | 1 | 1 | 1 | 0 |
| Non-crop agriculture | 0 | 1 | 1 | 2 | 2 | 2 | 2 | 2 | 1 | 1 |
| Open natural | 1 | 2 | 2 | 2 | 2 | 2 | 2 | 1 | 1 | 1 |
| Developed | 1 | 1 | 1 | 1 | 2 | 2 | 1 | 1 | 1 | 1 |
| Shrubland | 1 | 2 | 2 | 2 | 1 | 1 | 1 | 2 | 1 | 0 |

# 4. Calibration

## 4.1. Calibration methods

The calibration of BEEHAVE focused on a subset of parameters of the model as described in the main manuscript. Nectar and pollen resource availabilities in the landscape were included in the calibration because they were based on uncertain estimates. We tested three scenarios addressing the uncertainty in the seasonality of nectar and pollen resource availability from forest and woodland land covers (Table S13). The categories of resource availabilities in non-crop land covers represent declining nectar and pollen resources with declining categories whereby category 4 represented the highest food availability corresponding to a mass flowering crop. We applied two scenarios with scenario A assuming that nectar and pollen availabilities are half of the higher category. In scenario B, a linear decline and nectar and pollen with resource category is assumed. In both cases, category 0 corresponds to no nectar and pollen availability (Table S14). In correspondence, gathering times for nectar and pollen were also tested using four distinct scenarios (Table S15). Scenario 1 assumes constant gathering times irrespective of resource category, using the BEEHAVE default assumptions about gathering times of nectar and pollen. Scenario 2 assumes that gathering times in the lowest resource category 1 are doubled compared to the highest resource availability in category 4 with intermediate values assumed for categories 2 and 3. In scenario 3, we assumed that gathering times are tripled in category 1 compared to category 4. Finally, scenario 4 represents the range of gathering times reported in the literature (Dosselli et al. 2016; Eckert et al. 1994; Schmid-Hempel and Schmid-Hempel 1987; Thompson et al. 2016; Winston 1987), assuming that shortest gathering times correspond to our high resource category and longest observed gathering times to the lowest resource category.

**Table S13.** Resource categories (see Table S11) applied in the calibration to patches with forest/ woodland land cover in late winter and early spring (February – April).

| Forest/woodland spring resource category scenario | | Feb | Mar | Apr |
|---|---|---|---|---|
| FS1: Initial/ medium | Nectar | 2 | 3 | 3 |
| | Pollen | 2 | 3 | 3 |
| FS2: Medium nectar/ high pollen | Nectar | 2 | 3 | 3 |
| | Pollen | 3 | 4 | 4 |
| FS3: High nectar and pollen | Nectar | 2 | 4 | 4 |
| | Pollen | 2 | 4 | 4 |

**Table S14.** Definitions of non-crop resource categories applied in the calibrations: nectar and pollen availabilities.

| Category | Resource availability scenario A | | Resource availability scenario B | |
|---|---|---|---|---|
| | **Nectar ($l/m^2$)** | **Pollen ($g/m^2$)** | **Nectar ($l/m^2$)** | **Pollen ($g/m^2$)** |
| 0 | 0 | 0 | 0 | 0 |
| 1 | $1.25 \times 10^{-4}$ | 0.125 | $2.5 \times 10^{-4}$ | 0.25 |
| 2 | $2.5 \times 10^{-4}$ | 0.25 | $5 \times 10^{-4}$ | 0.5 |
| 3 | $5 \times 10^{-4}$ | 0.5 | $7.5 \times 10^{-4}$ | 0.75 |
| 4 | $1 \times 10^{-3}$ | 1 | $1 \times 10^{-3}$ | 1 |

**Table S15.** Definitions of non-crop resource categories applied in the calibrations: nectar and pollen gathering times.

| Cate-gory | Gathering time scenario I | | Gathering time scenario II | | Gathering time scenario III | | Gathering time scenario IV | |
|---|---|---|---|---|---|---|---|---|
| | **Nectar gather-ing time (s)** | **Pollen gather-ing time (s)** | **Nectar gather-ing time (s)** | **Pollen gather-ing time (s)** | **Nectar gather-ing time (s)** | **Pollen gather-ing time (s)** | **Nectar gather-ing time (s)** | **Pollen gather-ing time (s)** |
| 0 | 0 | 0 | 0 | 0 | 0 | 0 | 0 | 0 |
| 1 | 1200 | 600 | 2400 | 1200 | 3600 | 1800 | 4200 | 2100 |
| 2 | 1200 | 600 | 2000 | 1000 | 2800 | 1400 | 3040 | 1520 |
| 3 | 1200 | 600 | 1600 | 800 | 2000 | 1000 | 1880 | 940 |
| 4 | 1200 | 600 | 1200 | 600 | 1200 | 600 | 720 | 360 |

### *4.2. Calibration results*

In Figure S1, BEEHAVE outputs from a simulation of a single colony for adult bee numbers and brood are graphed along with corresponding CCA data. Figure S2 depicts the honey and pollen stores in BEEHAVE compared to CCA data from the same example colony. Qualitative discrepancies are marked in the figures and were observed consistently across all simulated control colonies from study LSCFS_2015_1.

In the BEEHAVE simulations prior to calibration, egg production by the queen ceased much earlier in the year than in the study colonies. This was expected because the seasonal egg-laying function applied in BEEHAVE was based on studies of bee colonies in Germany and Great Britain. Climatic conditions are very different in North Carolina where the LSCFS were conducted. Shorter winters result in longer periods when foraging and brood raising is possible. High peak adult bee numbers and high honey stores simulated in BEEHAVE pointed to overestimation of resource availability in the landscape, or too-low foraging effort in collecting the resources. In addition, low pollen stores suggested that the balance between foraging effort for nectar and pollen was not reflective of foraging in the study colonies.

However, pollen stores are highly variable and may differ considerably between colonies. In addition, pollen stores are measured with low accuracy in visual CCAs. Accordingly, pollen stores were not used to assess model fit to data. Visual assessments of brood numbers, particularly eggs and larvae, also provide relatively low accuracy. Attempts to increase the match between simulated and brood numbers reported from CCAs resulted in declining fit of adult bee numbers after initial adjustments. This suggests that it may be difficult to fit all simulation measures equally well to study data. In the following, calibration efforts were focused on achieving an acceptable fit between simulations and study data with respect to adult bee numbers and honey stores because these two measures were also identified as most important indicators of colony health, particularly prior to overwintering.

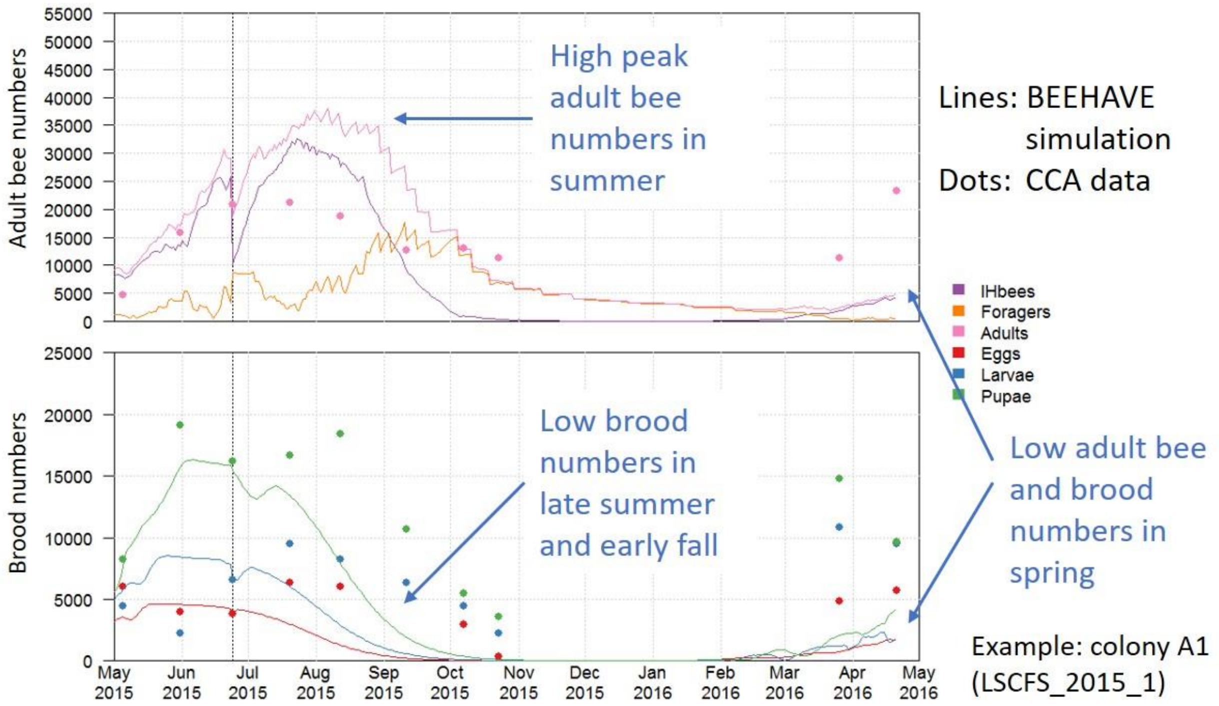


**Figure S1.** Adult bee and brood numbers in an example BEEHAVE simulation prior to calibration. CCA data from the corresponding colony are included as dots. The dotted vertical line denotes the reset of the BEEHAVE colony to conditions observed in the study colony at time of study initiation. Note that 'Adults' show the sum of 'IHbees' (in-hive bees) and 'Foragers'. Blue arrows and text emphasize qualitative deviations of BEEHAVE simulations from CCA data.

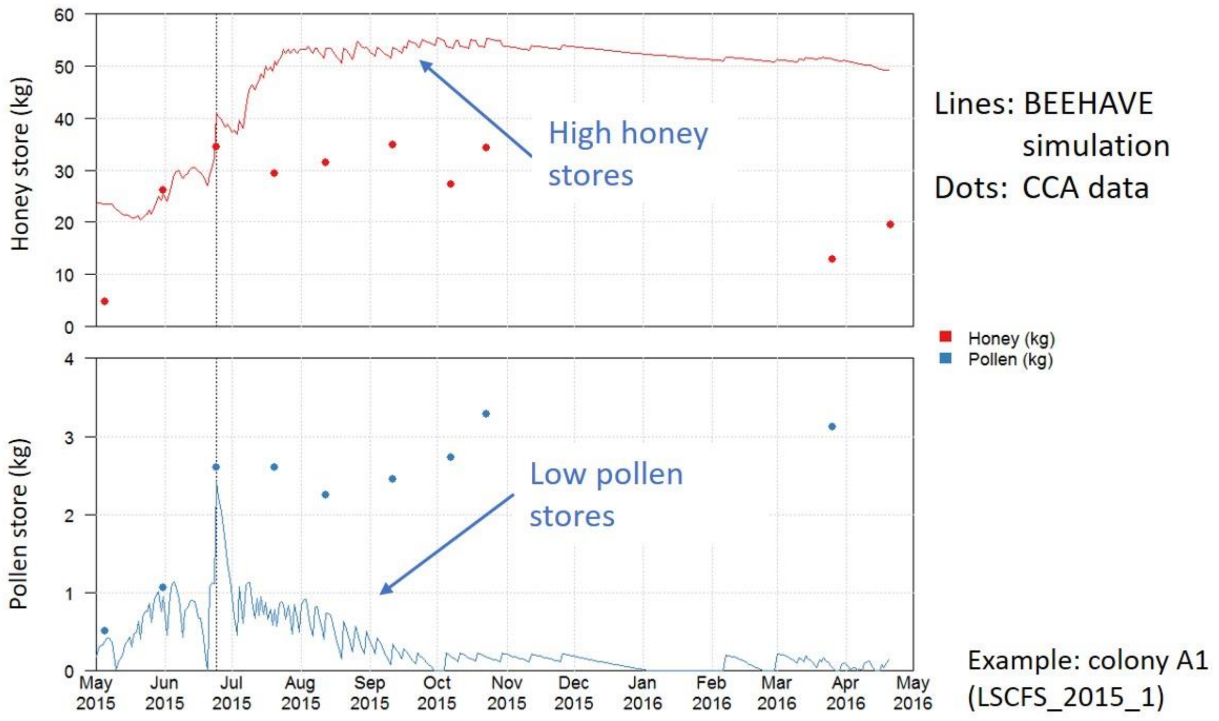


**Figure S2.** Honey and pollen stores (kg) in an example BEEHAVE simulation prior to calibration. CCA data from the corresponding colony are included as dots. The dotted vertical line denotes the reset of the BEEHAVE colony to conditions observed in the study colony at time of study initiation. Blue arrows and text emphasize qualitative deviations of BEEHAVE simulations from CCA data.

Testing several combinations of the parameters included in the calibration effort resulted in increased match between simulations and CCA data in one measure while reducing the match in another measure. We concluded that a good match across all measures and dates available for comparison may not be attainable. Subsequently, the calibration effort was focused on achieving a good match in adult bee numbers and honey stores. These two measures were identified as most important measures of colony health and had the lowest uncertainty in data reported from the studies. Fall was identified as particularly important time of the year because colony condition prior to overwintering is related to subsequent overwinter survival (Abi-Akar et al., submitted as companion paper).

The parameter combination that resulted in the best match between apiary-specific simulations and CCA data with the uncertainty range applied is summarized in Table 3. The decision on the set of parameter values was based on all apiaries from studies LSCFS_2015_1 and LSCFS_2015_2. For the purpose of the calibration, the determination of best match between simulations and CCA data ranges was conducted by graphing apiary-specific outcomes. In addition, the number of mean BEEHAVE measures that fell within the apiary-specific CCA data ranges for the fall (late October) CCA were compared between several sets of simulations. In Figure S3 and Figure S4, the comparison between BEEHAVE simulations and CCA data ranges are summarized for studies LSCFS_2015_1 and LSCFS_2015_2,

respectively. Note that during the calibration process, apiary-specific comparisons were conducted, i.e., data from 23 apiaries and corresponding simulations were compared individually. One apiary from study LSCFS_2015_2 was removed from comparison due to reported damage to the colonies in the apiary during the course of the study.

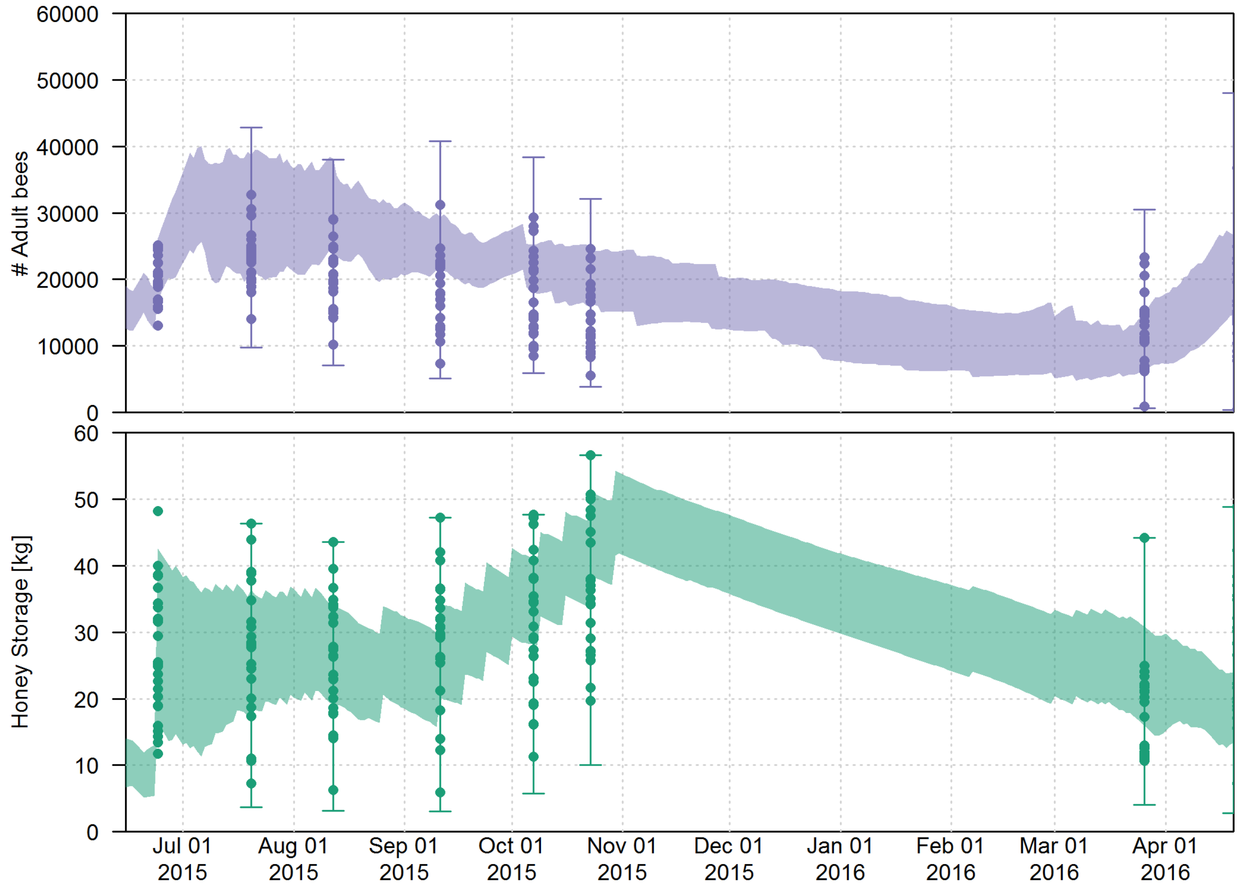


**Figure S3.** Adult bee numbers (top) and honey stores (bottom) in study LSCFS_2015_1 used for BEEHAVE calibration. Shaded areas show range of BEEHAVE outputs across all apiaries simulated. Dots represent the data reported from the CCAs. Lines with whiskers mark the range of CCA data across all apiaries and with uncertainty range applied to observations.

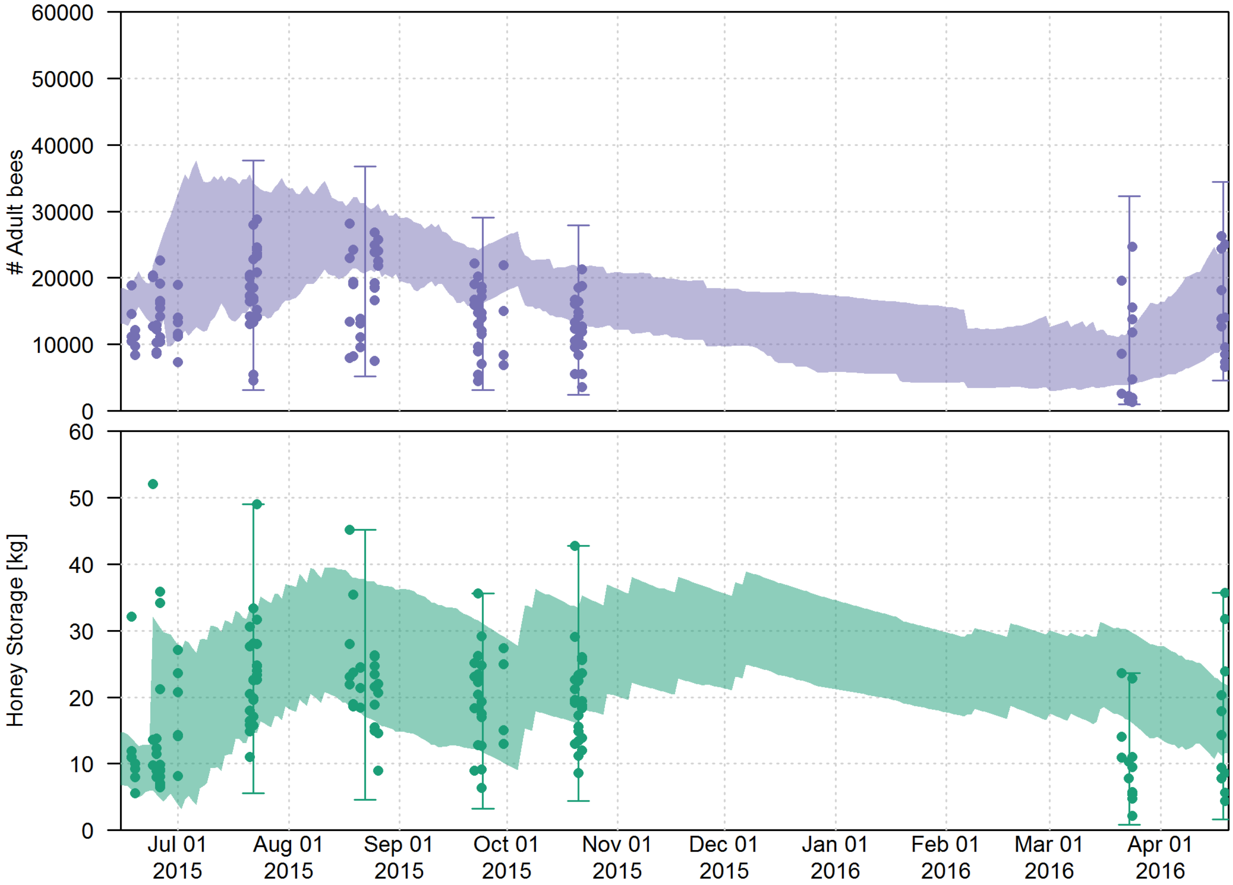


**Figure S4.** Adult bee numbers (top) and honey stores (bottom) in study LSCFS_2015_2 used for BEEHAVE calibration. Shaded areas show range of BEEHAVE outputs across all apiaries simulated. Dots represent the data reported from the CCAs. Lines with whiskers mark the range of CCA data across all apiaries and with uncertainty range applied to observations.

## References

Babendreier, D., Kalberer, N., Romeis, J., Fluri, P. and Bigler, F. 2004. Pollen consumption in honey bee larvae: a step forward in the risk assessment of transgenic plants. Apidologie 35, 293-300.

Bagavathiannen, M.V. 1999. Feral nature of alfalfa (Medicago sativa L.): implications for novel trait confinement. PhD Thesis, Department of Plant Science, University of Manitoba, Winnipeg, MB, Canada.

Becher, M.A., Grimm, V., Thorbek, P., Horn, J., Kennedy, P.J., Osborne, J.L. 2014. BEEHAVE: a systems model of honeybee colony dynamics and foraging to explore multifactorial causes of colony failure. Journal of Applied Ecology 51, 470-482.

Becher, M.A., Grimm, V., Knapp, J., Horn, J., Twiston-Davies, G., Osborne, J.L. 2016. BEESCOUT: A model of bee scouting behaviour and a software tool for characterizing nectar/pollen landscapes for BEEHAVE. Ecological Modelling 340, 126–133.

Brouwers, E.V.M., 1983. Activation of the Hypopharyngeal Glands of Honeybees in Winter. Journal of Apicultural Research 22, 137–141.

Cane, J., Gardner, D., Harrison, P.  2011. Nectar and pollen sugars constituting larval provisions of the alfalfa leaf-cutting bee (Megachile rotundata) (Hymenoptera: Apiformes: Megachilidae). Apidologie, 42, 401-408.

Carter, P.R., Hicks, D.R., Oplinger, E.S., Doll, J.D., Bundy, L.G., Schuler, R.T., Holmes, B.J. 1989. Grain Sorghum (Milo). Alternative Field Crops Manual. https://www.hort.purdue.edu/newcrop/afcm/sorghum.html

Cheung A.Y., Wu H.-M., Di Stilio V., Glaven R., Chen C., Wong E., Ogdahl J., Estavillo A. 2000. Pollen-pistil interactions in Nicotiana tabacum. Annals of Botany 85 (Supplement A): 29-37.

Chiari, W.C., Toledo, V. de A.A. de, Ruvolo-Takasusuki, M.C.C., Attencia, V.M., Costa, F.M., Kotaka, C.S., Sakaguti, E.S., Magalhães, H.R. 2005. Floral biology and behavior of Africanized honeybees Apis mellifera in soybean (Glycine max L. Merril). Brazilian Archives of Biology and Technology 48, 367–378.

Dittberner, P.L., Olson, M.R. 1983. The plant information network (PIN) data base: Colorado, Montana, North Dakota, Utah, and Wyoming. FWS/OBS-83/86. Washington, DC: U.S. Department of the Interior, Fish and Wildlife Service. 786 p.

Dosselli, R., Grassl, J., Carson, A., Simmons, L.W., Baer, B., 2016. Flight behaviour of honey bee (Apis mellifera) workers is altered by initial infections of the fungal parasite Nosema apis. Scientific Reports 6, 36649.

Eckert, C.D., Winston, M.L., Ydenberg, R.C., 1994. The relationship between population size, amount of brood, and individual foraging behaviour in the honey bee, Apis mellifera L. Oecologia 97, 248–255.

Frankl, R., Wanning, S., Braun, R. 2005. Quantitative floral phenology at the landscape scale: is a comparative spatio-temporal description of “flowering landscapes” possible? Journal for Nature Conservation 13, 219–229.

Gerik, T., Bean, B., Vanderlip, R. 2003. Sorghum growth and development. Texas Cooperative Extension. The Texas A&M University System. B-6137. amarillo.tamu.edu/files/2010/11/sorghum_growth_development.pdf

Hines, H.M., Hendrix, S.D. 2005. Bumble bee (Hymenoptera: Apidae) diversity and abundance in tallgrass prairie patches: effects of local and landscape floral resources. Environmental Entomology 34, 1477–1484.Hines and Hendrix 2005.

Horner, M., Mott, R.L. 1979. The frequency of embryogenic pollen grains is not increased by in vitro anther culture in Nicotiana tabacum L. Planta 147: 156-158.

Imdorf A, Buehlmann G, Gerig L, Kilchenmann V, Wille H. 1987. Überprüfung der Schätzmethode zur Ermittlung der Brutfläche und der Anzahl Arbeiterinnen in freifliegenden Bienenvölkern. Apidologie 18:137–146.

Jarosz, N., Loubet, B., Durand, B., Foueillassar, X., Huber, L. 2005. Variations in maize pollen emission and deposition in relation to microclimate. Environmental Science & Technology 39: 4377-4384.

Koti, S., Reddy, K.R., Reddy, V.R., Kakani, V. G., Zhao, D. 2004. Interactive effects of carbon dioxide, temperature, and ultraviolet-B radiation on soybean (Glycine max L.) flower and pollen morphology, pollen production, germination, and tube lengths. J Exp Bot 56: 725-736.

Lonsdorf, E., Kremen, C., Ricketts, T., Winfree, R., Williams, N., Greenleaf, S. 2009. Modelling pollination services across agricultural landscapes. Ann Bot 103, 1589–1600.

Oberhauser, K.S., Prysby, M.D., Mattila, H.R., Stanley-Horn, D.E., Sears, M.K., Dively, G., Olson, E., Pleasants, J.M., Lam, W.-K.F., Hellmich, R.L. 2001. Temporal and spatial overlap between monarch larvae and corn pollen. PNAS 98, 11913-11918.

Odoux, J.-F., Feuillet D., Aupinel, P., Loublier, Y., Tasei, J.-N., Mateescu, C. 2012. Territorial biodiversity and consequences on physico-chemical characteristics of pollen collected by honey bee colonies. Apidologie 43, 561-575

Palmer, R.G., Albertsen M.C., Heer, H. 1978. Pollen production in soybeans with respect to genotype, environment, and stamen position. Euphytica 27, 427-433.

Prieto-Baena, J.C., Hidalgo P.J., Dominguez, E., Galan, C. 2003. Pollen production in the Poaceae family. Grana 42: 153-159.

Reddi, C.S., Reddi, N.S. 1986. Pollen production in some anemophilous angiosperms. Grana 25:55-61.

Schmickl T, Crailsheim K. 2007. HoPoMo: A model of honeybee intracolonial population dynamics and resource management. Ecol Modell 204:219-245.

Schmid-Hempel, P., Schmid-Hempel, R., 1987. Efficient Nectar-Collecting by Honeybees II. Response to Factors Determining Nectar Availability. Journal of Animal Ecology 56, 219–227.

Southwick, E.E. 1984. Photosynthate allocation to floral nectar: a neglected energy investment. Ecology, 65: 1775-1779.

Thieret, J.W., Niering, W.A., Olmstead, N.C. 2001. National Audubon Society field guide to North American wild flowers. Eastern region. Second edition. Alfred A. Knopf, New York.

Thompson, H., Coulson, M., Ruddle, N., Wilkins, S., Harkin, S. 2016. Thiamethoxam: Assessing flight activity of honeybees foraging on treated oilseed rape using radio frequency identification technology. Environ Toxicol Chem, 35: 385–393.

Uribellarea, M., Carcova, J., Otegui, M.E., Westgate, M.E. 2002. Crop physiology & metabolism. Pollen production, pollination dynamics, and kernel set in maize. Crop Science 42: 1910-1918.

USDA (US Department of Agriculture). 2015. Attractiveness of agricultural crops to pollinating bees for the collection of nectar and/or pollen. Washington, DC.

USEPA (US Environmental Protection Agency). 2014. Guidance for Assessing Pesticide Risks to Bees. https://www.epa.gov/pollinator-protection/pollinator-risk-assessment-guidance

Winston ML. 1987. The biology of the honey bee. Harvard University Press, Cambridge, Massachusetts.
